# Uncovering two neutrophil-committed progenitors that immediately precede promyelocytes during human neutropoiesis

**DOI:** 10.1038/s41423-025-01259-w

**Published:** 2025-02-13

**Authors:** Ilaria Signoretto, Federica Calzetti, Giulia Finotti, Silvia Lonardi, Camillo Balanzin, Francisco Bianchetto-Aguilera, Sara Gasperini, Elisa Gardiman, Monica Castellucci, Anna Russignan, Massimiliano Bonifacio, Antonio Sica, William Vermi, Cristina Tecchio, Patrizia Scapini, Nicola Tamassia, Marco A. Cassatella

**Affiliations:** 1 Department of Medicine, Section of General Pathology, University of Verona, Verona, Italy; 2 Centro Piattaforme Tecnologiche, University of Verona, Verona, Italy; 3 Department of Molecular and Translational Medicine, Unit of Pathology, University of Brescia, Brescia, Italy; 4 Department of Engineering for innovation medicine, University of Verona, Verona, Italy; 5 Department of Pharmaceutical Sciences, University of Piemonte Orientale ‘A. Avogadro’, Novara//Humanitas Clinical and Research Center, Rozzano, Italy

**Keywords:** Neutrophil progenitors, Neutrophils, Neutropoiesis, Chronic myeloid leukemia, G-CSF, Neutrophils, Myelopoiesis

## Abstract

Technological advances have greatly improved our knowledge of myelopoiesis, for example, with the discovery of granulocyte‒monocyte‒dendritic cell (DC) progenitors (GMDPs), monocyte‒DC progenitors (MDPs), common DC progenitors (CDPs) and common monocyte progenitors (cMoPs) on the basis of flow cytometry approaches. Concomitantly, some progress has been made in characterizing the very early phases of human neutropoiesis with the description of novel CD66b^+^ progenitors, including eNePs, PMs w/o eNePs, ProNeus, and PreNeus. More recently, we identified four SSC^lo^Lin^-^CD66b^-^CD45^dim^CD34^+^/CD34^dim/-^CD64^dim^CD115^-^ cells as the earliest precursors specifically committed to the neutrophil lineage present in human bone marrow (BM), which we called neutrophil-committed progenitors (NCPs, from NCP1s to NCP4s). In this study, we report the isolation and characterization of two new SSC^hi^CD66b^-^CD64^dim^CD115^-^NCPs that, by phenotypic, transcriptomic, maturation and immunohistochemistry properties, as well as by flow cytometric side-scattered light (SSC), stand after NCP4s but precede promyelocytes during the neutropoiesis cascade. Similar to SSC^lo^CD45RA^+^NCP2s/NCP3s and SSC^lo^CD45RA^-^NCP1s/NCP4s, these cells exhibit phenotypic differences in CD45RA expression levels and, therefore, were named SSC^hi^CD45RA^+^NCP5s and SSC^hi^CD45RA^-^NCP6s. Moreover, NCP5s were more immature than NCP6s, as determined by cell differentiation and proliferative potential, as well as by transcriptomic and phenotypical features. Finally, by examining whether NCPs and all other CD66b^+^ neutrophil precursors are altered in representative hematological malignancies, we found that, in patients with chronic-phase chronic myeloid leukemia (CP-CML), but not with systemic mastocytosis (SM), there is an increased frequency of BM NCP4s, NCP6s, and all downstream CD45RA-negative neutrophil progenitors, suggesting their expansion in CML pathogenesis. Taken together, our data advance our knowledge of human neutropoiesis.

## Introduction

According to knowledge recently acquired by scRNA-seq studies and clonal tracking analysis [1, 2], hematopoiesis is currently believed to occur as a continuous process along developmental trajectories, without going through discrete hierarchic progenitor populations [3, 4]. In this context, the use of cutting-edge technologies has also expanded our knowledge of neutropoiesis, especially in relation to very immature neutrophil precursors, which likely correspond to the generically defined CD34^+^/CD34^+/dim^/CD34^-^ myeloblasts, but that have never been correctly characterized [4]. Accordingly, we recently identified CD34^+^ and CD34^dim/-^SSC^lo^CD66b^-^CD64^dim^CD115^-^ cells as the earliest precursors specifically committed to the neutrophil lineage present in human bone marrow (BM) [5]. These cells, named neutrophil-committed progenitors (NCPs) and able to generate neutrophils upon adoptive transfer into humanized mice, are subdivided into four populations on the basis of their differential expression of CD34 and CD45RA: CD34^+^CD45RA^-^NCP1s, CD34^+^CD45RA^+^NCP2s, CD34^dim/-^CD45RA^+^NCP3s and CD34^dim/-^CD45RA^-^NCP4s [5]. Furthermore, we also provided evidence that NCPs are more immature than other previously reported CD66b^+^ neutrophil precursors, including CD66b^+^CD15^+^CD49d^+^CD11b^-^ ProNeus [6, 7] and Lin^-^CD66b^+^CD71^+^CD117^+^eNePs and CD66b^+^CD71^+^CD117^-^PMs without eNePs (PMs w/o eNePs), which, according to Hedrick’s group [8, 9], form CD66b^+^promyelocytes (PMs).

Herein, we hypothesized that, by progressively increasing the number of intracellular granules and related content, CD34^dim/-^CD45RA^-^NCP4s might develop into more mature neutrophil precursors. Because the latter process is known to increase the side-scattered light (SSC) parameter [10] detected by flow cytometry, we decided to very carefully dissect the SSC^hi^CD66b^-/^^+^ cells within the low-density cells of the human BM (BM-LDCs). By doing so, we report the identification and characterization of previously undescribed SSC^hi^CD66b^-^NCPs, which we named NCP5s and NCP6s, both of which are more mature than the previously described CD34^+^ and CD34^dim/-^SSC^lo^CD66b^-^CD64^dim^CD115^-^NCPs, but more immature than the CD66b^+^PMs. Moreover, we report that CD34^dim/-^SSC^lo^CD66b^-^CD64^dim^CD115^-^NCPs, as well as SSC^hi^CD66b^-^NCPs, abnormally accumulate in BM samples from patients with chronic-phase chronic myeloid leukemia (CP-CML) but not with systemic mastocytosis (SM).

## Results

### Identification of novel Lin^-^SSC^hi^CD66b^-^CD11b^-^CD16^-^CD64^dim^CD115^-^CD117^+^CD71^hi^ neutrophil precursors within BM-LDCs

**Fig. 1:**
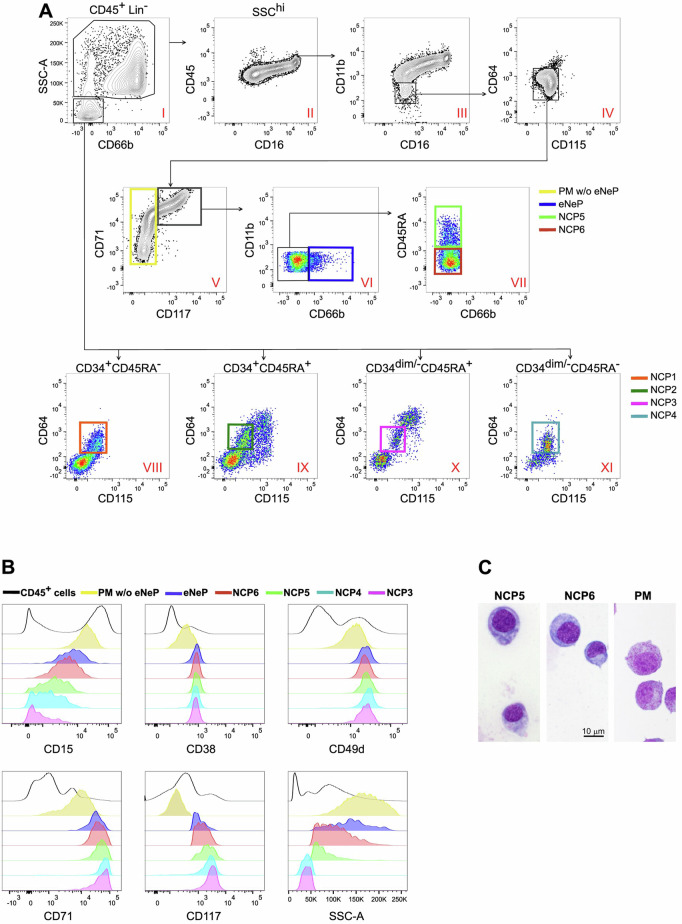
Identification of NCP5s and NCP6s in BM-LDCs. **A** Flow cytometry gating strategy illustrating how to identify Lin^-^SSC^hi^CD66b^-^CD11b^-^CD16^-^CD64^dim^CD115^-^CD117^+^CD71^hi^CD45RA^+^NCP5 (panel VII, light green gate) and Lin^-^SSC^hi^CD66b^-^CD11b^-^CD16^-^CD64^dim^CD115^-^CD117^+^CD71^hi^CD45RA^-^NCP6 (panel VII, red gate) within the SSC^hi^CD45^+^ cells in BM-LDCs (panel I), after the exclusion of mature CD16^-^eosinophils (panel II), mature CD11b^+^neutrophils (panel III), immature CD64^+^monocytes (panel IV), CD117^-^CD71^dim/hi^PM w/o eNePs (panel V, yellow gate) and CD71^+^CD117^+^CD66b^+^eNePs (panel VI, blue gate). The bottom panels show the identification of SSC^low^CD66b^-^CD34^+^CD64^dim^CD115^-^CD45RA^-^ NCP1s (panel VIII, orange gate), SSC^low^CD66b^-^CD34^+^CD64^dim^CD115^-^CD45RA^+^ NCP2s (panel IX, green gate), SSC^low^CD66b^-^CD34^dim/-^CD64^dim^CD115^-^CD45RA^+^ NCP3s (panel X, magenta gate) and SSC^low^CD66b^-^CD34^dim/-^CD64^dim^CD115^-^CD45RA^-^ NCP4s (panel XI, light blue gate). One representative experiment (out of 10 performed, with similar results) is shown. **B** Histograms depicting the expression of CD15, CD38, CD49d, CD71, and CD117, as well as that of SSC-A, by total CD45^+^BM-LDC cells, NCP3s, NCP4s, NCP5s, NCP6s, eNePs and PMs w/o eNePs, as defined in panel (**A**). The data are representative of 1 of 5 independent experiments, with similar results. **C** Morphology of purified NCPs and PMs. Sorted NCP5s, NCP6, and PMs were stained via the May-Grunwald procedure

To analyze the phenotype of the SSC^hi^CD66b^-/^^+^ cells present within human BM-LDCs, we assembled an antibody panel that, via flow cytometry, stains key markers of recently identified neutrophil precursors, including (i) CD64, CD115, and CD45RA for NCPs [5]; (ii) CD71 and CD117 for Lin^-^CD66b^+^CD71^+^CD117^+^eNePs [8, 9]; and (iii) CD66b, CD16, and CD11b for PMs, myelocytes (MYs), metamyelocytes (MMs), band cells (BCs), and segmented neutrophils (SNs) [11]. As shown in Fig. S1A, mature lymphoid and monocytic cells, as well as CD123^+^ cells [which include dendritic cell (DC) progenitors and mature plasmacytoid DCs (pDCs)], were excluded from our analysis (Fig. S1A, panel IV and panel V) to focus on both mature and immature granulocytes (Fig. S1A, panel V, alias Fig. 1A, panel I). By doing so, we confirmed that the previously described NCP1-4s stand within the SSC^lo^BM-LDCs (as gated in panels VIII, IX, X, and XI of Fig. 1A) [5], which, therefore, from hereafter will be collectively referred to as SSC^lo^NCPs. By analyzing the SSC^hi^CD66b^+^BM-LDCs and SSC^hi^CD66b^-^BM-LDCs (Fig. 1A, panel I), after excluded mature CD45^hi^CD16^low^ eosinophils (Fig. 1A, panel II) and subsequently gated the CD11b^-^CD16^-^ cells (Fig. 1A, panel III), which included conventional PMs (Fig. S1B, middle panel), we ultimately focused on the CD64^dim^CD115^-^ cells (Fig. 1A, panel IV). Interestingly, we divided the latter cell populations into CD117^+^CD71^hi^ and CD117^-^CD71^dim/hi^cells on the basis of their CD71 and CD117 expression (Fig. 1A, panel V). The CD117^-^CD71^dim/hi^cells were all CD66b^+^ (Fig. S1B), while the CD117^+^CD71^hi^ cells were further subdivided into CD66b^-^ and CD66b^+^ cells (Fig. 1A, panel VI), with the CD117^+^CD71^hi^CD66b^+^ cells corresponding to the previously described eNePs [9]. Consequently, we concluded that the CD117^-^CD71^dim/hi^ cells (Fig. 1A, panel V), which represent the majority of conventional PMs depleted of CD66b^+^CD117^+^CD71^hi^eNePs, correspond to the CD66b^+^CD71^dim/ hi^CD117^-^PMs w/o eNePs described by Dinh et al. [9].

**Fig. 2:**
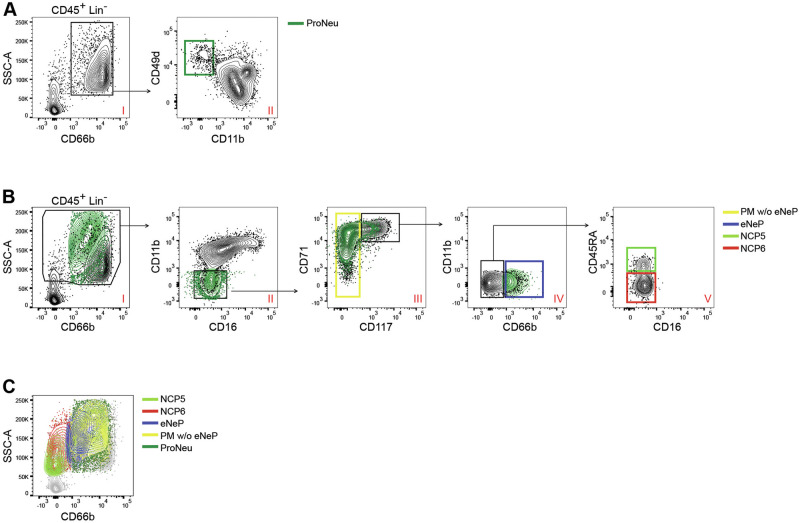
Phenotypic comparison of ProNeus with NCP5s, NCP6s, eNePs, and PMs w/o eNePs by flow cytometry. **A** Flow cytometry gating strategy to identify the ProNeus in SSC^hi^Lin^-^CD45^+^ cells in BM-LDCs (panel II, green gate) according to their CD66b^+^CD49d^+^CD11b^-^ phenotype. **B** Plots showing how ProNeus (green dots as defined in **A**) overlay PMs w/o eNePs (yellow gate), eNePs (blue gate), NCP5s (light green gate) and NCP6s (red gate), as defined by the gating strategy illustrated in Fig. 1. **C** Contour plot overlays highlighting the differences among NCP5s, NCP6s, eNePs, PM w/o eNePs and ProNeus in terms of the SSC-A parameter and CD66b expression. The data are representative of 1 of 4 independent experiments, with similar results

We next focused on the CD66b^-^cell populations, which were predicted to be neutrophil progenitors standing at a maturation level positioned between SSC^lo^NCPs and eNePs, which we renamed SSC^hi^CD66b^-^NCPs because of their Lin^-^CD64^dim^CD115^-^ phenotype and high SSC properties. Characterization of the SSC^hi^CD66b^-^NCPs revealed that they are heterogeneous in terms of CD45RA expression (Fig. 1A, panel VII), with the CD45RA^+^ cell population being less represented (0.14 ± 0.02% of the total CD45^+^ cells, *n* = 10) than the more abundant CD45RA^-^ cell population (0.73 ± 0.05%). Furthermore, because of their Lin^-^SSC^hi^CD66b^-^CD11b^-^CD16^-^CD64^dim^CD115^-^CD117^+^CD71^hi^ CD45RA^+^ and CD45RA^-^ phenotypes, which recapitulate the CD45RA^+^NCP3s and CD45RA^-^NCP4s, respectively, we renamed the SSC^hi^CD45RA^+^ cells NCP5s (Fig. 1A, panel VII) and the SSC^hi^CD45RA^-^ cells NCP6s (Fig. 1A, panel VII). A detailed analysis of NCP5s and NCP6s, compared with NCP3s, NCP4s, eNePs and PMs w/o eNePs, in terms of the expression levels of markers associated with neutropoiesis revealed that while CD15 gradually increases from NCP3s to PMs w/o eNePs, CD117 concomitantly and progressively decreases (Fig. 1B). In contrast, while CD38, CD49d, and CD71 levels were found to remain elevated in all neutrophil progenitors, with a tendency to decline in PMs w/o eNePs (Fig. 1B), the SSC parameter was found to increase starting from both NCP5s and NCP6s, being expressed at the highest levels by PMs w/o eNePs. Morphologically, NCP5s and NCP6s display variable sizes, round eccentric nuclei, a basophilic cytoplasm, and visible granules; the latter are less homogeneously distributed than those observed in PMs (Fig. 1C)_._ To further contextualize NCP5s and NCP6s in relation to the human SSC^hi^CD66b^+^CD15^+^ CD49d^+^CD11b^-^ ProNeus [6, 7], which, according to the recently proposed nomenclature of CD66b^+^neutrophil progenitors, represent the most immature progenitors [downstream followed by the preNeus, ImmatureNeus and MatureNeus [6]], anti-CD49d antibodies were added to our staining panel. As shown in Fig. 2, we selected the ProNeus within the SSC^hi^CD66b^+^ cells of BM-LDCs according to their phenotype (Fig. 2A, panel II) and, in turn, overlaid them on the gating strategy used to identify NCP5s and NCP6s (Fig. 2B). As expected, ProNeus overlapped with the SSC^hi^CD66b^+^fraction of neutrophil progenitors (Fig. 2B, panel I) and resulted in a CD11b^-^CD16^-^ phenotype, i.e., a phenotype corresponding to conventional CD66b^+^CD11b^-^CD16^-^PMs (Fig. 2B, panel II). Accordingly, ProNeus also included both CD66b^+^CD71^hi^CD117^+^eNePs and CD66b^+^CD71^dim/hi^CD117^-^PMs w/o eNePs (Fig. 2B, panels III and IV) but not CD66b^-^CD11b^-^CD16^-^CD71^hi^CD117^+^CD45RA^+^NCP5s or CD11b^-^CD16^-^CD71^hi^CD117^+^CD45RA^-^NCP6s (Fig. 2B, panels IV-V). In support of these observations, an overlay of SSC^hi^CD66b^-^NCPs, ProNeus, eNePs and PMs w/o eNePs on an SSC versus CD66b plot shows that, starting from NCP5s and ending at the PM w/o eNePs, neutrophil precursors gradually increase surface CD66b, which very neatly distinguishes NCP5s and NCP6s from CD66b^+^ProNeus, CD66b^+^eNePs and CD66b^+^PM w/o eNePs (Fig. 2C). As expected [12] ^(Fig. 137)^, our experiments demonstrated that ProNeus phenotypically overlapped with both eNePs and PMs w/o eNePs.

### Lin^-^SSC^hi^CD66b^-^CD11b^-^CD16^-^CD64^dim^CD115^-^CD117^+^CD71^hi^CD45RA^+^NCP5s and Lin^-^SSC^hi^CD66b^-^CD11b^-^CD16^-^CD64^dim^CD115^-^CD117^+^CD71^hi^CD45RA^-^NCP6s represent neutrophil precursors

**Fig. 3:**
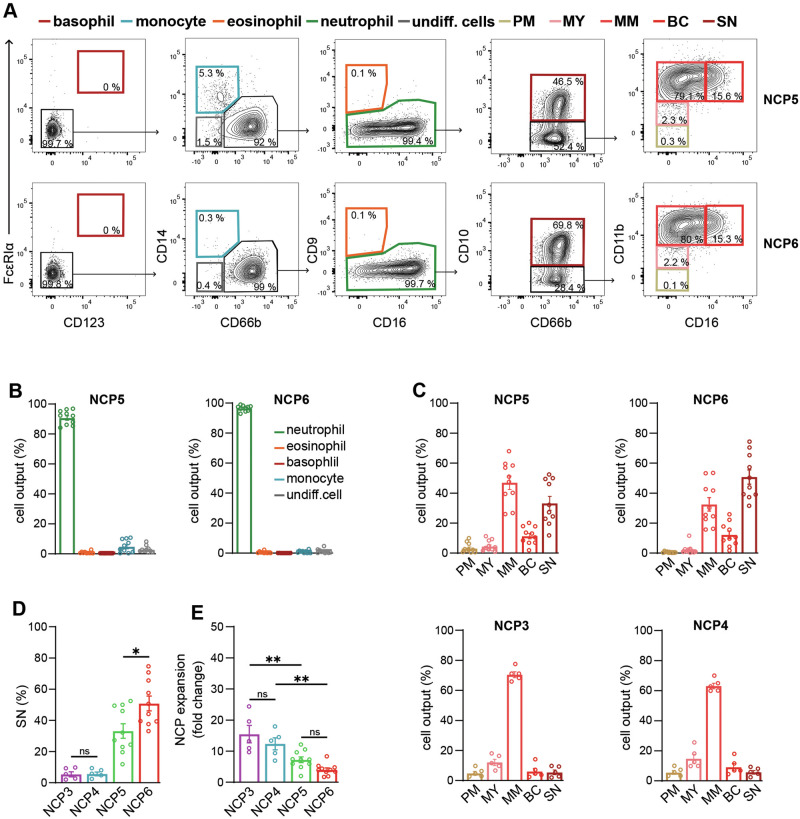
Differentiation of NCP5s and NCP6s into CD66^+^ cells. **A** Representative flow cytometry gating strategy used to analyze CD66b^+^ cells derived from NCP5s and NCP6s cultured with SFGc for 5 days (*n* = 10). Plots showing the identification of basophils (bordeaux gate), monocytes (light blue gate), eosinophils (orange gate) and undifferentiated cells (gray gate), as well as PMs (beige gate), MYs (pink gate), MMs (light red gate), BCs (red gate) and SNs (dark red gate) within the CD66b^+^ cells (green gate). **B** Bar graphs showing the percentages of CD66b^+^ cells (green contour, mean ± s.e.m. refers to total CD45^+^ cells, *n* = 10), eosinophils (orange contour), basophils (bordeaux contour), monocytes (light blue contour) and undifferentiated cells (gray contour) derived from NCP5s and NCP6s cultured for 5 days with SFGc. **C** Bar graphs showing the percentages of CD66b^+^PMs (beige contour), MYs (pink contour), MMs (light red contour), BCs (red contour) and SNs (dark red contour) derived from NCP3s, NCP4s, NCP5s and NCP6s cultured for 5 days with SFGc (mean ± s.e.m., *n* = 5 for NCP3s and NCP4s; *n* = 10 for NCP5s and NCP6s). **D** Bar graph representing the generation of SNs (alias CD10^+^ cells) from NCP3s, NCP4s, NCP5s and NCP6s treated with SFGc for 5 days (mean ± s.e.m., *n* = 5 for NCP3s and NCP4s; *n* = 10 for NCP5s and NCP6s). **E** Bar graph displaying the fold expansion of purified NCP3s, NCP4s, NCP5s and NCP6s treated with SFGc for 5 days (mean ± s.e.m., *n* = 5 for NCP3s and NCP4s; *n* = 10 for NCP5s and NCP6s). **D**, **E** Statistical analysis was performed via one-way ANOVA and Tukey’s post hoc test. * = *p* < 0.05, ** = *p* < 0.01, *** = *p* < 0.001

To establish that NCP5s and NCP6s effectively represent neutrophil-restricted progenitors, they were sorted according to the gating strategy depicted in Fig. S1C and then cultured in the presence of SFGc on an MS-5 cell layer, as previously described for SSC^lo^NCPs [5]. Initial experiments revealed that the viability of the cells generated by both NCP5s and NCP6s was significantly greater on day 5 (94.4 ± 2.4% for NCP5s and 93.1 ± 2.2% for NCP6s) than on day 7 (80.2 ± 9.1% for NCP5s and 69.6 ± 12.6% for NCP6s), unlike the cells generated by sorted NCP3s and NCP4s (and used as terms of reference) (Fig. S1D). Hence, NCP5s and NCP6s treated with SFGc for 5 days mostly differentiated into CD66b^+^ cells (89.4 ± 4.4% from NCP5s, 96.3 ± 2.0% from NCP6s, *n* = 8) and, exclusively in the case of NCP5s, into a small number of CD14^+^ monocytes (5.9 ± 3.9%, *n* = 8; Fig. 3A, B). The fact that some monocytes could be recovered as the cell output of SFGc-treated NCP5s is not surprising since, as previously observed for SFGc-treated NCP2s and NCP3s [5, 13], CD45RA^+^ neutrophil progenitors (such as NCP2s, NCP3s and NCP5s) could be minimally contaminated by monocyte precursors unavoidably cosorted owing to their CD64 and CD45RA expression.

Phenotypic analysis of the CD66b^+^ cells derived from SFGc-treated NCP5s and NCP6s revealed that these cells belong to the neutrophil lineage only, as no eosinophils were detected on the basis of CD9 and CD16 expression (Fig. 3A, B), unlike in the case of control BM-LDCs (Fig. S1E). Moreover, CD66b^+^ cells derived from NCP5s and NCP6s were found to stand at different stages of maturation (Fig. 3C), predominantly consisting of CD66b^+^CD10^-^CD11b^dim^CD16^-^MYs, CD66b^+^CD10^-^CD11b^+^CD16^dim^MMs, CD66b^+^CD10^-^CD11b + CD16+ BCs and CD66b^+^CD10^+^CD11b^+^CD16^++^SNs, in any case at a relatively higher maturation grade than the CD66b^+^ cells (consisting of MMs mainly) derived from NCP3s and NCP4s (Fig. 3C, bottom panels). Notably, NCP6s were found to generate significantly more SNs than NCP5s (Fig. 3D), indicating that they are more mature. Moreover, even though NCP5s and NCP6s collectively presented lower proliferation rates than did NCP3s and NCP4s (Fig. 3D), NCP5s and NCP3s were more proliferative than were NCP6s and NCP4s, respectively (Fig. 3E).

To further support their unilineage commitment, we incubated both NCP5s and NCP6s (with NCP3s and NCP4s as controls) in the presence of the SFG cocktail (containing GM-CSF instead of G-CSF) for 5 days. Under SFG treatment, both NCP5s and NCP6s were found to mostly differentiate into CD66b^+^ cells that displayed the typical CD66b^+^CD10^-^CD11b^dim^CD16^-^ phenotype of MYs (Fig. S2B). Notably, more CD66b^+^ cells were detected from NCP6 (86.4 ± 5.9%, n = 8) than from NCP5s (68.6 ± 11.9%), as well as fewer undifferentiated cells (Fig. S2A). In contrast, NCP3s and NCP4s were found to generate fewer CD66b^+^ cells (46.7 ± 3.2% from NCP3s, 52.6 ± 14.9% from NCP4s, *n* = 3), predominantly consisting of CD66b^+^CD10^-^CD11b^-^CD16^-^ PMs (Fig. S2A, B). By measuring their proliferative capacity, we also observed that both NCP5s and NCP6s expanded less than did NCP3s and NCP4s under SFG treatment, with NCP5s manifesting greater proliferative capacities than NCP6s (Fig. S2C). In these experiments, although the overall expansive capacity of all NCPs was found to significantly decrease with respect to SFGc (compare Fig. S3C with Fig. 3E), differences in the capacity to expand between NCP5s and NCP3s, as well as between NCP4s and NCP6s, were maintained (Fig. S2C).

In subsequent experiments, we investigated the respiratory burst activity, phagocytosis capacity, and cytokine production ability of CD66b^+^ cells generated by both NCP5s and NCP6s after 5 days of culture with SFGc (Fig. S3). Confirming their higher grade of maturity, the CD66b^+^ cells generated by both NCP5s and NCP6s were found to release greater amounts of superoxide anions in response to PMA than those generated by NCP3s and NCP4s (Fig. S3A). Consistently, CD66b^+^ cells generated by both NCP5s and NCP6s were found to phagocytose unopsonized zymosan, similar to HD neutrophils (Fig. S3B), as well as to produce and release CXCL8, IL-1ra and BAFF (Fig. S3C). In the latter case, we observed that in response to LPS, CD66b^+^ cells generated by both NCP5s and NCP6s released CXCL8 and IL-1ra at higher levels than HD neutrophils did (Fig. S3C), which might be explained by the fact that G-CSF amplifies the production of both cytokines in neutrophils [14, 15]. Our hypothesis is supported by the fact that only the CD66b^+^ cells generated by NCP5s and NCP6s, but not those generated from HDs, constitutively release BAFF (Fig. S3C), which is in line with our previous observations in G-CSF-treated donors [14].

Finally, we evaluated the ability of donor-derived HSCs to repopulate NCP5s and NCP6s in the BM of allo-HSTC transplant patients. For this purpose, LD-BMCs were isolated from BM aspirates collected from the iliac crest on day 21 after transplantation and then analyzed via flow cytometry to identify NCPs. As shown in Fig. S3D, we detected donor-derived NCP5s and NCP6s among the reconstituted cells, including mature neutrophils, in the BM of patients. These findings indicate that NCP5s and NCP6s are derived from donor-derived HSCs and that, in turn, they contribute to the generation of mature neutrophils in an in vivo human model.

### RNA-seq experiments confirmed that NCP5s and NCP6s precede conventional PMs during neutropoiesis

**Fig. 4:**
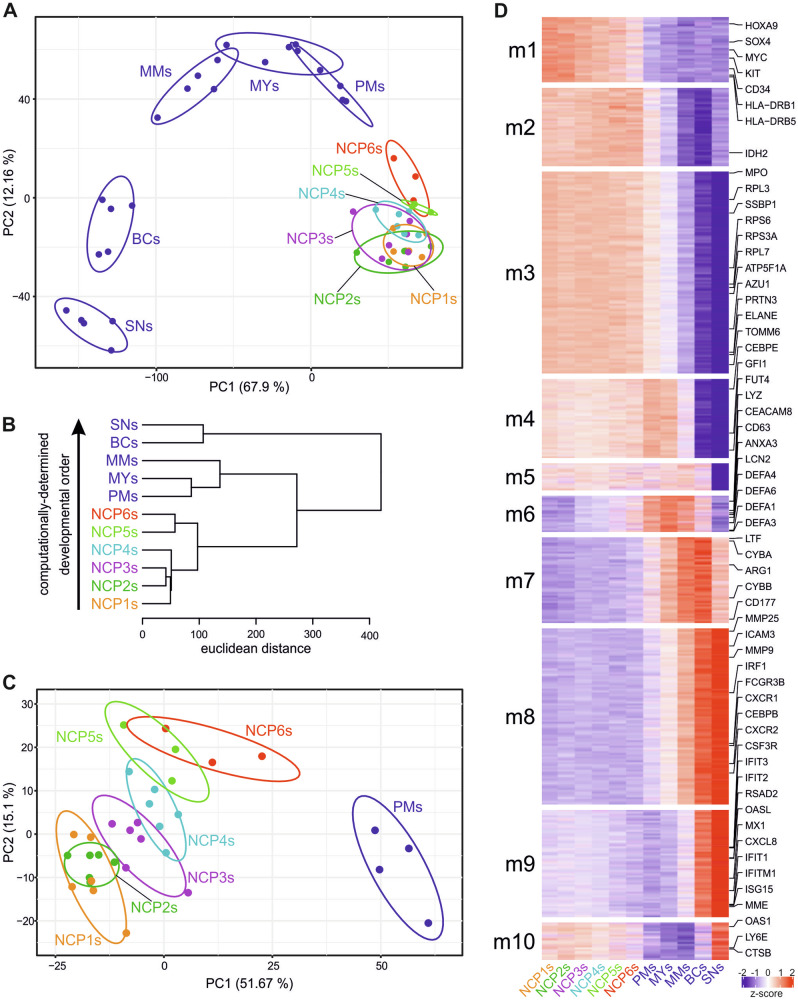
RNA-seq experiments revealed that NCP5s and NCP6s precede conventional PMs during neutropoiesis. **A** PCA scatter plot based on the DEGs identified from bulk RNA-seq analyses of NCP5s (light green) and NCP6s (red) as well as NCP1s (orange), NCP2s (green), NCP3s (magenta) and NCP4s (turquoise), PMs, MYs, MMs, BCs and SNs (*n* = 3–6). **B** Developmental paths of NCPs and other CD66b^+^ cells computationally determined from bulk RNA-seq datasets via the optimal leaf ordering (OLO) algorithm. **C** PCA scatter plot (as in panel **A**) focused exclusively on all NCPs and PMs. **D** Heatmap displaying the expression patterns of the gene modules (m1–m10) resulting from the k-means analysis of DEGs identified among the various neutrophil-lineage cells. The median gene expression levels of the biological replicates were calculated, and the data were represented as z scores. The relevant genes for each module are listed on the right

Studies utilizing bulk gene expression profiling techniques, including microarray [16] and RNAseq [17], have shown that transcriptome analyses represent effective methods for determining the degree of maturation of putative neutrophil progenitors. Hence, we profiled NCP5s and NCP6s, as well as NCP1-4 s, conventional PMs, MYs, MMs, BCs, and SNs, via bulk RNA-seq. To identify differentially expressed genes (DEGs) across the various samples, we applied likelihood ratio tests (LRTs) and obtained a total of 6900 DEGs. PCA of these DEGs revealed that, on average, NCP5s clustered with NCP1-4 s more closely than with NCP6s did and that NCP6s preceded conventional PMs (Fig. 4A) along the neutrophil maturation trajectory, as confirmed by hierarchical clustering analysis performed via optimal leaf ordering (OLO) (Fig. 4B). To more precisely illustrate the transcriptomic differences among the NCPs only, we performed a new PCA specifically focused on all NCPs and PMs (Fig. 4C). This new analysis revealed that NCP1s and NCP2s cluster closely together, being separated from NCP3s and even more from NCP4s. Along the same maturation trajectory, both NCP5s and NCP6s are clearly separated, thus confirming their differences at the transcriptomic level. By performing K-means clustering of the DEGs, ten gene modules (m1–m10) were identified among all the samples (Fig. 4D and Table S3). Their analysis revealed that, similar to NCP1s-NCP4s but distinct from conventional PMs or downstream neutrophil precursors, NCP5s and NCP6s express genes belonging to m1, which is enriched in genes typically associated with immature cells, such as *HOXA9*, *MYC*, *SOX4*, and *KIT* (Fig. 4D and Table S3). Unlike conventional PMs and/or downstream neutrophil precursors, NCP5s and NCP6s also express high levels of m2 and m3 genes, which include not only mRNAs encoding azurophilic granule (AG) proteins but also those involved in ribosome assembly and mitochondria formation, two biological processes associated with very immature neutrophil stages [17, 18]. NCP5s and NCP6s, together with NCP1s-NCP4s, PMs, MYs, and MMs, also express m4 and m5 genes, including neutrophil-specific transcription factors (i.e., *CEBPE* and *GFI1*) and typical neutrophil membrane markers (i.e., *FUT4*/CD15). In contrast, m6, including genes encoding defensins (*DEFA1*, *DEFA3*, and *DEFA4* being the most highly expressed), bactericidal proteins (such as *LYZ* and *BPI*) and discrete neutrophil markers (such as *CEACAM8*/CD66b and *CD63*), were found in NCP5s/NCP6s even though they were maximally expressed at the PM, MY, and MM stages. On the other hand, m7-m10 genes, including mRNAs encoding gelatinase granule (GG) proteins (i.e., *MMP9*, *ARG1, CD177* and *CTSB*), interferon-stimulated genes (ISGs), NADPH oxidase components (i.e., *CYBA* and *CYBB*), and various receptors (i.e., *FCGR3B*, *FPR1*, *FPR2*, *CXCR1* and *CXCR2*), were found to be mostly expressed in mature neutrophils but not (or at very low levels) in NCP5s and NCP6s. In summary, comparative transcriptomic analysis of NCP5s and NCP6s with other neutrophil precursors clearly revealed NCP5s and NCP6s immediately after SSC^lo^NCPs and prior to conventional PMs during neutropoiesis.

### The mRNA expression profiles of granule proteins function as distinctive criteria for identifying NCP5s and NCP6s

**Fig. 5:**
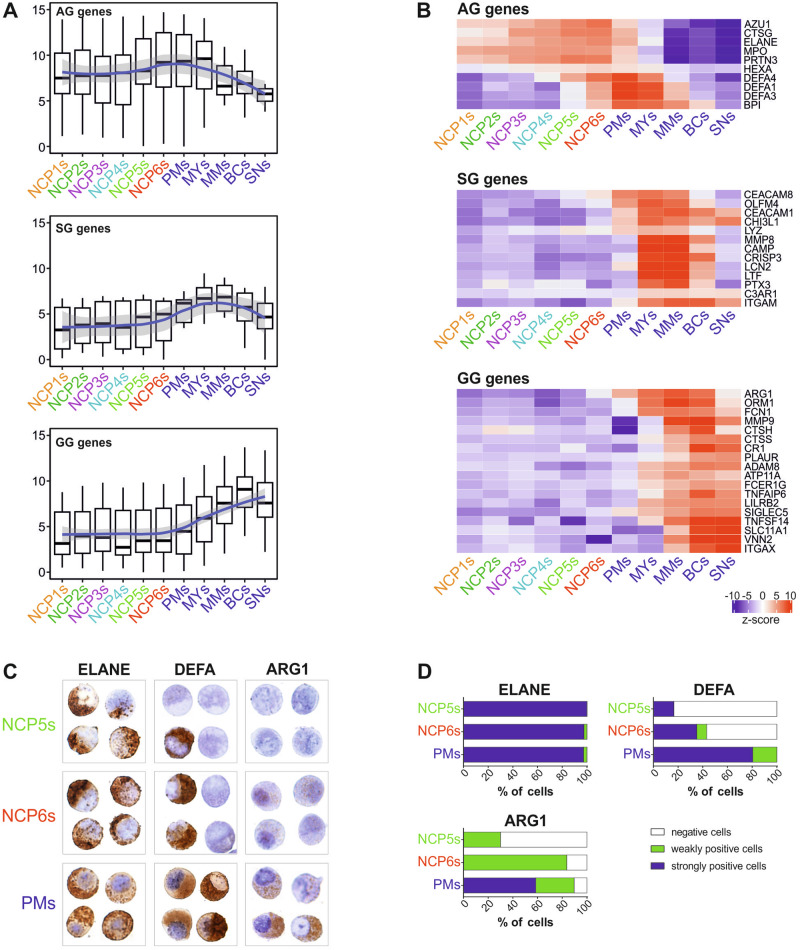
Transcriptional and immunocytochemical analyses of the expression profiles of representative azurophilic, specific, and gelatinase granule proteins in NCP5s and NCP6s. **A** Box plots showing the distribution of mRNA expression levels [log2(FPKM + 1)] of genes associated with AG, SG and GG. The box plot shows the median with the lower and upper quartiles representing the 25th to 75th percentile range and whiskers extending to the 1.5 × interquartile range (IQR). LOESS fitting of the data with a relative confidence interval is represented by a blue line with a shadow area. **B** Heatmaps showing the expression levels of selected genes encoding typical azurophilic, specific, and gelatinase granule proteins. The median gene expression levels of the biological replicates (*n* = 3--6) were calculated, and the data were represented as z scores. **C**, **D** Expression of antigenic elastase (ELANE), α-defensins (DEFAs) and arginase-1 (ARG1) by immunocytochemical staining of NCP5s, NCP6s and PMs. **C** Four representative stained cells for each cell population are shown. Original magnification, ×600. **D** Stacked bar graph displaying the percentage of cells showing negative (white), weakly positive (green), or strongly positive (blue) immunocytochemical staining for ELANE, DEFA and ARG1 by specific antibodies

By analyzing the mRNA expression levels of granule proteins in all our samples, we found that the expression of AG genes was elevated in SSC^lo^NCPs, peaked in NCP6s, PMs, and MYs, and subsequently declined (Fig. 5A). However, a more detailed analysis based on their transcript levels revealed that AG genes can be divided into two groups (Fig. 5B, top panel): (i) a group including the *AZU1*, *CTSG*, *ELANE*, *MPO* and *PRTN3* genes, which are already expressed in both NCP1s/NCP2s, progressively increase in both NCP3s/NCP4s, reach their maximal expression in NCP5s and NCP6s and then gradually decrease; (ii) a second group that instead includes defensins and *BPI* genes which, even if present in AG, are expressed starting from NCP5s and NCP6s, reach their maximal expression in PMs and then decrease at later stages of neutrophil maturation. We observed that the majority of the SG genes was not expressed in SSC^lo^NCPs (Fig. 5A, middle panels, and B), whereas only a few (such as *CEACAM8*, *OLFM4* and *ARG1*) started to be transcribed in NCP5s and NCP6s (Fig. 5B, middle panel and bottom panels). Nonetheless, SG genes were found to reach their maximal expression at the MY and MM stages and then to decrease in more mature stages (Fig. 5A, B, middle panels). Finally, we found that GG genes are absent in all NCPs, including NCP5s and NCP6s, but, as previously described [17, 19], they start to be transcribed at the MY stage, reaching their maximal expression in BCs (Fig. 5A, B, bottom panels).

By immunocytochemistry (ICC) experiments, we found that both NCP5s and NCP6s were positive for elastase (Fig. 5C, D), and that NCP5s (16%), and at relatively high levels (35%) NCP6s, started to express α-defensins (Fig. 5C, D). In the latter case, we reported that NCP1s/NCP2s are completely negative for defensins and that NCP3s/NCP4s display a low percentage of weakly positive cells [5], in contrast to the 80% conventional PMs found to strongly express α-defensins (Fig. 5C, D). Arginase, which is commonly associated with GG proteins, was expressed (albeit at low levels) in NCP5s and (more) in NCP6s (Fig. 5C, D), which is in line with the mRNA data. In contrast, arginase was positive in all PMs, with over 50% of them displaying strong positivity (Fig. 5C, D). Overall, these data confirm that the process of neutrophil differentiation is intricately linked to the kinetics of granule protein synthesis. According to our data, both NCP5s and NCP6s result in the neutrophil differentiation stage in which α-defensins start to be expressed at both the mRNA and protein levels.

### NCP6s are derived either directly from NCP4s or indirectly from NCP3s via NCP5s

**Fig. 6:**
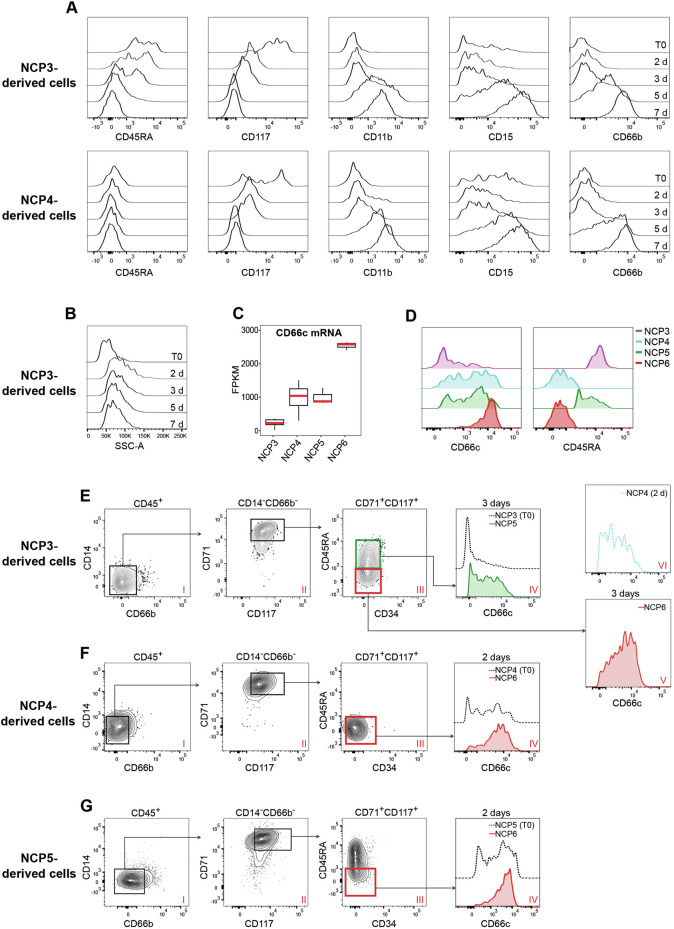
Evidence for the ability to generate both NCP5s and NCP6s via NCP3s and NCP6s via NCP4s. Flow cytometry histograms displaying the CD45RA, CD117, CD11b, CD15, and CD66b expression levels (**A**), as well as the SSC-A parameter (**B**), of the cells derived from NCP3s and NCP4s cultured with SFGc for 0, 2, 3, 5, and 7 days. The data are representative of 1 out of 4 independent experiments performed with similar results. **C** Box plots illustrating CD66c mRNA expression levels in NCP3s (*n* = 6), NCP4s (*n* = 6), NCP5s (*n* = 3), and NCP6s (*n* = 3). The box plot shows the median with the lower and upper quartiles representing the 25th to 75th percentile range and whiskers extending to the 1.5X interquartile range (IQR). **D** Flow cytometry histograms showing CD66c and CD45RA expression by NCP3s, NCP4s, NCP5s, and NCP6s within BM-LDCs. One representative experiment out of 4 is shown. **E** Flow cytometry plots displaying the generation from NCP3s of CD66b^-^CD71^+^CD117^+^CD45RA^+^CD66c^-/+^NCP5s (green gate and histogram filled in green), CD66b^-^CD71^+^CD117^+^CD45RA^-^CD66c^++^NCP6s (red gate and histogram filled in red) after 3 days, and CD66b^-^CD71^+^CD117^+^CD45RA^-^CD66c^+/-^ NCP4s (histogram with dotted line in light blue) after 2 days. Flow cytometry plots displaying the generation of CD66b^-^CD71^+^CD117^+^CD45RA^-^CD66c^++^ NCP6s from either NCP4s (**F**) or NCP5s (**G**) after 2 days of culture with SFGc. Panels IV of (**E**–**G**) display CD66c expression (dotted black lines) by freshly isolated (T0) NCP3s, NCP4s and NCP5s used as internal controls. **E–G** Representative data from 3 experiments with similar results are shown

Next, we investigated which progenitors NCP5s and NCP6s originate from. Previously, we showed that NCP4s are derived either directly from NCP1s or indirectly from NCP2s *via* NCP3s. We thus concluded that NCP3s must directly mature into NCP4s to subsequently generate PMs [5]. Thus, the identification of NCP5s as SSC^hi^CD66b^-^CD45RA^+^ cells is inconsistent with our previous conclusions about NCP3s and consequently suggests that additional maturation pathways are likely involved. Consistently, we also showed that the full downregulation of CD45RA expression by CD66b-CD45RA^+^NCP3s to become PMs (98.9 ± 0.1% (*n* = 10) of which are CD45RA^-^ cells), starts after 2 days and is fully completed after 7 days of culture in SFGc [5]. Therefore, we hypothesized that (i) there might be a fraction of NCP3s that, by maintaining CD45RA expression, generate NCP5s, which, in turn, generate NCP6s by downregulating CD45RA, and (ii) NCP4s can directly generate NCP6s. To test whether our hypotheses were correct, we sorted both NCP3s and NCP4s and cultured them on MS-5 cells with SFGc for 2, 3, 5, and 7 days before harvesting the generated cells for flow cytometry analysis. As expected [5], SFGc-treated NCP3s were found to differentiate into CD66b^+^ cells by gradually downregulating CD45RA and CD117 and, conversely, by expressing conventional neutrophil lineage markers such as CD11b, CD15, and CD66b (Fig. 6A, top panels). In contrast, NCP4s never expressed CD45RA during maturation (thus proving that they could never become NCP5s) but rather gradually expressed conventional neutrophil lineage markers (Fig. 6A, bottom panels).

Next, since the increase in SSCs detected in neutrophil progenitors directly gated from freshly obtained BM-LDCs was not mirrored by those that matured in vitro (please compare Fig. 6B with Fig. 1B), we took advantage of the CD66c marker to clearly distinguish NCP5s and NCP6s generated in vitro from the more immature NCPs. In fact, starting from NCP3s and NCP4s, CD66c (encoded by the *CEACAM6* gene) was found to be differentially expressed between NCP3s and NCP4s/NCP5s and NCP6s at both the mRNA (Fig. 6C) and protein levels (Fig. 6D, left panel). Moreover, in combination with CD45RA, CD66c was also found to discriminate CD45RA^+^CD66c^-^NCP3s from CD45RA^-^CD66c^+/-^NCP4s and CD45RA^+^CD66c^+/–^NCP5s from CD45RA^-^CD66c^++^NCP6s (Fig. 6D, right panel). Therefore, on the basis of CD66c and CD45RA expression, we demonstrated that the cells generated from NCP3s on day 3 were CD66b^-^CD11b^-^CD71^+^CD117^+^ and either CD45RA^+^ or CD45RA^-^ (Fig. 6A and panel III of Fig. 6E). The CD45RA^+^ fraction was found to display a phenotype compatible with CD45RA^+^CD66c^+/-^NCP5s (panel IV of Fig. 6E), whereas the CD45RA^-^ fraction was compatible with the CD45RA^-^CD66c^++^phenotype recalling NCP6s (panel V of Fig. 6E). Importantly, the cells generated from NCP3s on day 2 displayed the CD45RA^-^CD66c^+/-^ phenotype, which resembled NCP4s (panel VI of Fig. 6E), whereas those generated from SFGc-treated NCP4s on day 2 corresponded to the CD45RA^-^CD66c^++^NCP6s (panel IV of Fig. 6F). Finally, upon treatment with SFGc for 2 days, NCP5s start to downregulate CD45RA and upregulate CD66c expression to ultimately acquire the CD45RA^-^CD66c^++^ phenotype of NCP6s (panel IV of Fig. 6G).

Collectively, these data show that NCP3s, in part, mature into CD45RA-NCP6s via NCP4s and, in part, maintain CD45RA expression to become NCP5s before ultimately converging into NCP6s and then to PMs. Finally, the data also show that freshly isolated NCP4s directly and exclusively mature into NCP6s.

### NCP4s and NCP6s accumulate in the BM of chronic-phase chronic myeloid leukemia (CP-CML) patients but not in those with systemic mastocytosis (SM)

**Fig. 7:**
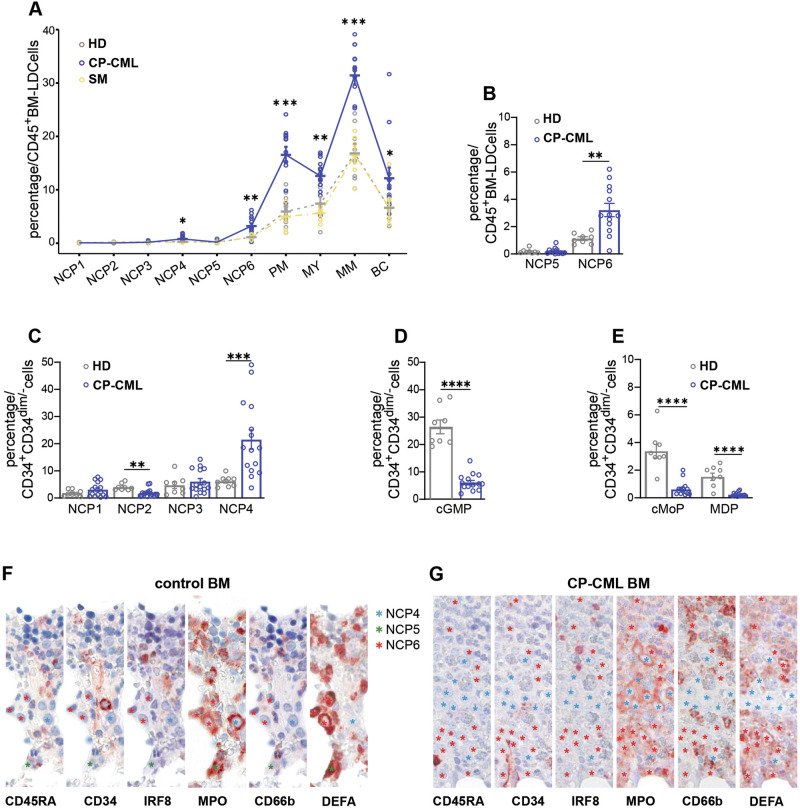
Flow cytometry and immunohistochemistry analysis of neutrophil progenitors in the BM-LDCs of HDs, CP-CML patients, and SM patients. **A** Frequencies of NCP1s, NCP2s, NCP3s, NCP4s, NCP5s, NCP6s, PMs, MYs, MMs and BCs in CD45^+^ bone marrow-low density cells (BM-LDCs) from HDs (*n* = 8, gray line), CP-CML patients (*n* = 13, blue line) and SM patients (*n* = 6, light yellow). **B** Bar graph highlighting NCP5s and NCP6s, as reported in panel (**A**), from HDs (gray contour) and CP-CML patients (blue contour) in CD45^+^BM-LDCs. **C** Bar graph showing the frequency of NCP1s, NCP2s, NCP3s, and NCP4s in the narrower area defined as CD34^+^/CD34^dim/-^ cells in HDs (*n* = 8, gray contour) compared with those in CP-CML patients (*n* = 15, blue contour). **D**, **E** Bar graph showing the frequency of cGMPs, cMoPs and MDPs in CD34^+^/CD34^dim/-^ cells from HDs (*n* = 8, gray contours) compared with those from CP-CML patients (*n* = 14, blue contours). **A**, **C**, **D** Data are presented as the means ± s.e.m.s. Statistical analysis was performed via the Mann‒Whitney test. * = *p* < 0.05, ** = *p* < 0.01, *** = *p* < 0.001, **** = *p* < 0.0001. Human FFPE BM sections from (**F**) HDs (*n* = 3) and (**G**) CP-CML patients (*n* = 3) were immunostained as indicated. Fewer neutrophil precursors (based on larger and round nuclei) are found in normal tissue than in CP-CML tissue. Numerous NCP4s and NCP6s are instead identifiable in BM CP-CML, as indicated by the colored asterisks (turquoise for NCP4, green for NCP5 and red for NCP6)

CP-CML is a chronic myeloproliferative disorder characterized by a dramatic increase in the number of peripheral white blood cells (WBCs) (i.e., higher than 10^10^/L), which mainly reflects a remarkable increase in the absolute number of neutrophils [20, 21], as also observed in our cohort of first-diagnosed patients investigated before therapy (Fig. S4A). In contrast, neutropoiesis is unaffected in SM, which is the most common form of mastocytosis diagnosed in adults; the latter disease results from the clonal proliferation of mast cells in extracutaneous organs, including the BM [22]. In fact, individuals affected by SM display a normal number of circulating neutrophils [23], as was also observed in our patient cohort (Fig. S4A). Therefore, we asked whether NCPs are detectable in the BM of CP-CML (or SM) patients and, if so, whether their frequency is altered. By utilizing the previously described gating strategies (Figs. 1A and 3), all NCPs and downstream PMs, MYs, MMs and BCs from both CP-CML (*n* = 13) and (as controls) SM (*n* = 6) patients could be identified unequivocally (Fig. 7A, B). Notably, the BM of CP-CML patients was found to contain, in general, greater numbers of neutrophil precursors than the BM of both SM patients and HDs (*n* = 8) (Fig. 7A, B). More specifically, the frequencies of NCP6s, PMs, MMs and BCs related to the total number of CD45^+^BM-LDCs in the BM of CP-CML patients were significantly greater than those in the BM of HDs or SM patients (Fig. 7A, B). Moreover, when the frequencies of SSC^lo^NCPs in the BM of CP-CML patients were related to CD34^+^CD34^dim/-^ cells, the number of SSC^lo^CD45RA^-^NCP4s was significantly greater than that in the BM of HDs (Fig. 7C) or SM patients (Fig. S4B). Importantly, we also confirmed previous observations [24, 25] on the significant decrease in cGMP from the BM of CP-CML patients compared with the BM of HDs (Fig. 7D, left panel) or SM patients (Fig. S4C, left panel), which we found to be accompanied by parallel, significantly diminished frequencies of monocyte progenitors (cMoPs) and monocyte‒dendritic progenitors (MDPs) (Fig. 7E, left panel; Fig. S4C, right panel). Even NCP2s were found to be significantly decreased in the BM of CP-CML patients (Fig. 7C), as expected given their inclusion within cGMPs [5].

For the final experiments, FFPE BM samples from HDs and CP-CML patients were subjected to consecutive immunostaining for CD45RA, CD34, IRF8, MPO, CD66b and α-defensins (DEFAs) to identify NCPs. IRF8 and MPO staining were used to replace CD115 and CD64, respectively, since the latter antigens could not be properly detected by IHC. In both BM samples from HDs and CP-CML patients (panels E, F of Fig. S4), we could clearly identify CD45RA^-^CD34^-^IRF8^-^MPO^+^CD66b^-^DEFA^-^, CD45RA^+^CD34^-^IRF8^-^MPO^+^CD66b^-^DEFA^+^, and CD45RA^-^CD34^-^IRF8^-^MPO^+^CD66b^-^DEFA^+^ cells that, according to our flow cytometry and ICC data (Figs. 1A, 5C, D), likely correspond to SSC^lo^CD45RA^-^NCP4s (light blue asterisks in panels F, G of Fig. 7), SSC^hi^CD45RA^-^NCP5s (green asterisks in panels F of Fig. 7) and SSC^hi^CD45RA^-^NCP6s (red asterisks in panels F, G of Fig. 7). Notably, both SSC^lo^CD45RA^-^NCP4s and SSC^hi^CD45RA^-^NCP6s, but not SSC^hi^CD45RA^-^NCP5s, were more abundant in the BM of CP-CML patients than in that of HDs (Fig. 7F, G), which is in line with the data shown in Fig. 7A–C. Interestingly, both NCP4s and NCP6s may be in direct contact with each other and form clusters in the BM section of the CP-CML patient (Fig. 7G). NCP4s under mitosis are also shown in panel F of Fig. S4.

## Discussion

**Fig. 8:**
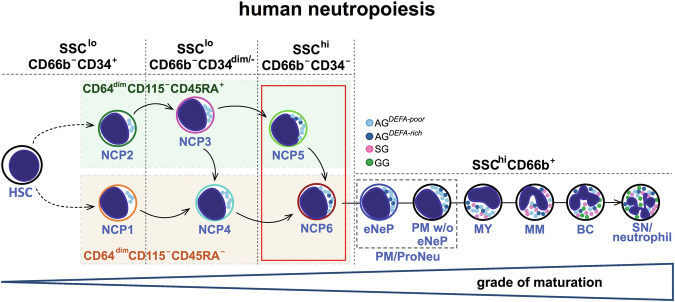
Our current model concerning the early phases of human neutropoiesis. The identification of SSC^hi^CD66b^-^CD45RA^+^NCP5s and SSC^hi^CD66b^-^CD45RA^-^NCP6s (boxed in red in the scheme) allows us to update our proposed model of human neutropoiesis [4]. At its very early stages, in fact, neutropoiesis includes SSC^lo^NCPs (i.e., NCP1s, NCP2s, NCP3s, NCP4s), then SSC^hi^NCPs (i.e., NCP5s and NCP6s), followed by eNePs and PM w/o eNePs (that form the PM), as depicted in the scheme. The arrows define the differentiation hierarchies of the individual NCPs to the PM

This study reports the identification and characterization of two new SSC^hi^CD66b^-^neutrophil-committed progenitors (NCPs), which we named NCP5s and NCP6s, which are consistent with our previously described SSC^lo^NCPs [5]. In fact, NCP5s and NCP6s were found to be either CD45RA^+^ or CD45RA^-^ cells, thus reflecting, respectively, CD34^+^CD45RA^+^NCP2s/CD34^dim/-^CD45RA^+^NCP3s and CD34^+^CD45RA^-^NCP1s/CD34^dim/-^CD45RA^-^NCP4s. By several criteria, we demonstrate that, during neutropoiesis, NCP5s and NCP6s follow NCP4s but immediately precede the CD66b^+^CD11b^-^CD16^-^PMs, as depicted by the scheme proposed in Fig. 8, which includes all recently reported CD66b^-^ and CD66b+ neutrophil progenitors. In fact, by standardizing our experimental settings with those from previous studies [9] and hence including anti-CD117 and anti-CD71 antibodies in our antibody panel, we confirmed not only that eNePs and the PM w/o eNePs form PMs {as proposed by Hedrick’s group [9]} but also that eNePs and the PM w/o eNePs express higher levels of neutrophil maturation markers (such as CD15 and CD66b) than NCP5s and NCP6s do. Moreover, by reproducing the criteria used by Ng and colleagues to classify neutrophil progenitors according to their CD49d expression [7], we confirmed recent data from the same Ng et al. group on the correspondence between SSC^hi^CD66b^+^CD15^+^CD49d^+^CD11b^-^ proNeus and conventional CD66b^+^CD11b^-^CD16^-^ PMs [12] ^(Fig. 137)^. However, since eNePs, PMs without eNePs and proNeus are all CD66b^+^ cells, they must cluster at later stages of neutropoiesis than CD66b^-^NCP5s and CD66b^-^NCP6s do. Moreover, in support of the notion that NCP5s and NCP6s are more mature than the SSC^lo^NCPs are a series of in vitro differentiation experiments demonstrating that, as predicted by their immunophenotype, after 5 days of culture in SFGc they generate CD66b^+^ cells at different stages of maturation undoubtedly more mature than those derived from NCP3s and NCP4s. Similar results were observed when SFG (which replaces G-CSF with GM-CSF) was used as a differentiation cocktail [5], although the generated CD66b^+^ cells were less differentiated and in lower numbers. Nonetheless, the unilineage commitment by NCP5s and NCP6s was, again, further supported. Notably, CD66b^+^ cells generated by NCP5s and NCP6s were found to function like HD neutrophils according to experiments evaluating their respiratory burst activity, phagocytosis capacity, and cytokine production ability.

We could then establish that SSC^hi^CD45RA^+^NCP5s are slightly more immature than SSC^hi^CD45RA^-^ NCP6s for the following reasons: (i) NCP5s display a lower SSC (likely reflecting a stage of insufficient granule production) and lower CD15 levels than NCP6s; (ii) NCP5s were found to expand slightly more than NCP6s in in vitro differentiation experiments, as well as to generate fewer segmented CD10^+^ neutrophils than NCP6s; and (iii) by immunohistochemistry, NCP5s contain less α-defensin and arginase than NCP6s do. The fact that NCP5s and NCP6s directly follow NCP4s, with NCP6 being slightly more mature than NCP5s and preceding the conventional PMs, was also demonstrated by the transcriptomic results. Accordingly, transcriptomic characterization of SSC^hi^CD66b^-^NCPs revealed a very high expression of AG genes (such as ELANE, MPO, and AZU1), as well as of α-defensins, compared with that of SSC^lo^NCPs. Notably, in past studies involving density gradient separation of neutrophil granules and electron microscopy, two types of AGs were observed on the basis of their content of α-defensins: defensin-rich and defensin-poor granules [26]. It was suggested that defensin-poor AGs are characteristic of less mature neutrophil progenitor stages, whereas defensin-rich AGs distinguish more mature neutrophil progenitor stages [27]. Our results align with this hypothesis, since antigenic α-defensins were detectable in NCP6s at higher levels than in NCP5s. Moreover, in a study in which we analyzed the gene expression profiles of neutrophil precursors from a patient with a nonsense mutation in SMARCD2, we detected a significant impairment in α-defensin mRNA expression, whereas the transcription of other AG proteins, such as MPO and ELANE, was normal [28]. In this context, neutrophils from patients with mutations in *CEBPE*, a transcription factor that interacts with SMARCD2 and is crucial for neutrophil maturation, also exhibit impaired expression of α-defensins [29]. On the basis of these two findings, we speculate that NCP5s and, more likely, NCP6s represent neutrophil progenitors in which SMARCD2 and CEBPE likely begin to function and promote the transcription of α-defensins and other SG and GG proteins, such as *LTF, LCN2* and *MMP8*.

In another series of experiments, we focused on identifying the neutrophil precursors generating SSC^hi^CD45RA^+^NCP5s and SSC^hi^CD45RA^-^NCP6s. Since the SSC parameter could not be exploited in in vitro differentiation experiments, we took advantage of the CD66c marker. In fact, together with CD45RA, CD66c expression was found to distinguish NCP3s from NCP4s, NCP4s from NCP5s, and NCP5s from NCP6s unequivocally. By doing so, we revealed that NCP3s, in part, mature into CD45RA^-^NCP6s via NCP4s and, in part, generate CD45RA^+^NCP5s by maintaining CD45RA expression. NCP5s, in turn, generate NCP6s by downregulating CD45RA. We also revealed that NCP4s, owing to their inability to upregulate CD45RA, can generate only NCP6s. Interestingly, we found that the progeny of NCP3s was composed of CD45RA^+^CD66c^+/-^NCP5s and CD45RA^-^CD66c^++^NCP6s after three days of culture, whereas the progeny of NCP4s was found to completely consist of CD45RA^-^CD66c^++^NCP6s after two days of culture. These types of kinetics support the hypothesis that the generation of NCP6s, while occurring directly from NCP4s, requires more complex steps from NCP3s, including their maturation into NCP4s. These findings support our previous data [5] on the existence of two differentiation pathways that, in the very early phases of neutropoiesis, converge to the PM (see Fig. 8): one that likely starts from CD45RA^-^NCP1s and that proceeds via NCP4s and NCP6s; the other, likely starting from CD45RA^+^NCP2s, proceeding *via* CD45RA^+^NCP3s and CD45RA^+^NCP5s, the latter originating from CD45RA^-^NCP6s. While the first pathway, even though unexpected given that it does not initiate from classic CD45RA^+^GMPs but rather from CD45RA^-^NCP1s [5], is much easier to comprehend, the second path is more complex because it assumes that NCP3s partly mature into NCP4s and partly into NCP5s. Our results indicate that the downregulation of CD45RA expression that occurs during neutrophil maturation is a much more complex and gradual process than assumed. The biological meaning(s) of the two maturation cascades converging into the PMs is still unknown. Since CD45RA^+^ cells tend to be more immature than CD45RA^-^ cells are, we speculate that the former cells are localized in hematopoietic niches that are distant from vessels, unlike the CD45RA^-^ cells, which we foresee to be promptly available for mobilization.

Finally, and not surprisingly, we detected a general expansion of neutrophil progenitors in BM samples from patients with CP-CML at first diagnosis and without treatment, but not from SMs, via flow cytometry experiments. More notably, and confirmed by immunostaining experiments, we found that such expansion starts at the level of the SSC^lo^CD45RA^-^NCP4s, involves the SSC^hi^CD45RA^-^NCP6s but not the SSC^hi^CD45RA^+^NCP5s, and continues through the entry of more differentiated neutrophil progenitors. Although these comparisons have been made between cohorts of different ages, these data are consistent with previous observations suggesting that the accumulation of neutrophil progenitors, which typically characterize CP-CML patients, specifically affects intermediate and later maturation compartments [30]. Further experiments are necessary to establish whether CML NCPs exhibit greater proliferative, survival, and differentiation capacity than HD NCPs do, as well as whether CML CD45RA^-^NCPs do the same versus CD45RA^+^ NCPs do. Nonetheless, the fact that it is possible to isolate and work on NCPs from pathological samples implies that, in the future, NCPs might be manipulated for potential therapeutic use.

## Materials and methods

### Isolation of cells from human bone marrow (BM)

HDs for BM samples (*n* = 21) were selected either by the Italian Bone Marrow Donor Registry (IBMDR) for unrelated recipients or by related donors of patients undergoing allogeneic transplants at our institution. Samples were obtained during BM harvest from the iliac crest upon Institutional Ethical Committee approval and patient informed consent. Importantly, none of our donors had undergone G-CSF treatment for BM donation. BM samples from CP-CML patients at first diagnosis and before therapy (*n* = 16), as well as from SM patients with BM involvement (*n* = 7) (Table S1 for patient characteristics), were obtained as part of diagnostic sampling upon local ethical committee approval. Briefly, the first 1.5–2 ml of fresh BM samples were collected under aseptic conditions in a heparinized sterile syringe, processed in endotoxin-free polypropylene tubes (Greiner bio-One, Kremsmüster, Austria), and subjected to density gradient centrifugation in low endotoxin Ficoll-Paque PLUS (Cytiva, Marlborough, MA, USA) to eliminate mature neutrophils and red cells. Hemodilution of BM samples was always excluded by verifying that the percentage of mature neutrophils never reached 20%. After centrifugation, BM low-density cells (BM-LDCs) were collected and either immediately processed for flow cytometry analysis or suspended in “culture medium” [αMEM growth medium (Corning Inc., Corning, NY, USA) supplemented with 10% low-endotoxin FBS ( < 0.5 EU/ml, Sigma‒Aldrich, St. Louis, MO, USA) and 1% pen/strep] to be finally distributed into tissue-6-well culture plates (Corning) at 10^7^ cells/ml and then [5] preincubated for 20 h at 37 °C under 5% CO_2_ before use, as previously described. For selected experiments, BM samples from patients subjected to alloHSTC were obtained on day +21 (*n* = 3), i.e., when hematopoiesis was actively reconstituting from the donor-derived HSCs.

### Flow cytometry and fluorescence-activated cell sorting experiments

For flow cytometry experiments, BM-LDCs were harvested, counted and resuspended in 100 μl of “staining buffer” [PBS (Corning) plus 2% FBS and 2 mM EDTA (Sigma‒Aldrich)] to be subsequently incubated for 10 min in the presence of 5% pooled, heat-inactivated, human serum (HS). Then, the cells were stained for 30 min on ice with fluorochrome-conjugated monoclonal antibodies (mAbs, listed in Table S3) and analyzed with an 18-color FACSAriaIII Fusion™ cell sorter (BD, Franklin Lakes, NJ, USA). For fluorescence-activated cell sorting of NCP3s, NCP4s, NCP5s, and NCP6s, 5–10 × 10^7^ BM-LDCs were resuspended at 10^8^/ml, labeled with fluorochrome-conjugated mAbs (listed in Table S2) for 45 min at 4 °C (in the dark), washed and resuspended in staining buffer at 3 × 10^7^/ml, and ultimately filtered through a 35-µm nylon mesh. The cells were finally sorted by using a FACSAriaIII Fusion™ cell sorter (BD) equipped with an 85-µm nozzle, immediately centrifuged, resuspended in αMEM, counted, and used for experiments. For differentiation experiments, cells generated from SFGc-treated NCP3s, NCP4s, NCP5s and NCP6s were harvested at the time points indicated in the text, resuspended in 50 μl of “staining buffer,” labeled with fluorochrome-conjugated mAbs (listed in Table S2) for 30 min at 4 °C (in the dark), and then analyzed with a MACSQuant16 Analyzer flow cytometer (Miltenyi Biotec, Bergisch Gladbach, Germany). FlowJo software v.10.10 was used for data analysis. For the selected experiments, a number of sorted cells were lysed in RLT buffer (Qiagen, Venlo, the Netherlands) for RNA extraction.

### In vitro differentiation assay and cell viability

To assess their differentiation potential, sorted NCP3s, NCP4s, NCP5s, and NCP6s were cultured on the MS-5 stromal cell line (ACC 441, Leibniz-Institut DSMZ, Braunschweig, Germany) according to established protocols [31]. Briefly, the day before the coculture experiments, MS-5 cells cultured in αMEM at 95% confluence were incubated with 10 µg/ml mitomycin C (Sigma‒Aldrich) for 3 h at 37 °C. After treatment with 0.05% trypsin/EDTA (Corning), MS-5 cells were collected and resuspended in αMEM at 0.25 × 10^6^/ml to be finally seeded in round-bottom 96-well tissue culture plates for 24 h. Then, 1–3 × 10^3^ sorted BM progenitors were resuspended in 100 μl of αMEM, seeded on top of MS5-containing cells and incubated, as previously described [5], with SFGc [i.e., a cocktail of 20 IU/ml FLT3L (Miltenyi Biotec), 10 IU/ml SCF (Miltenyi Biotec), and 6500 IU/ml G-CSF (Myelostim, Italfarmaco Spa)] or SFG [FLT3L, SCF, and 100 IU/ml GM-CSF (Miltenyi Biotec)]. Cells derived from neutrophil progenitors were then harvested from the MS-5 cells at the time points indicated in the text and then prepared for flow cytometry staining. The viability of BM-LDCs and NCP-derived cells was assessed via SYTOX Blue and the SytoxAADvanced Dead Cell Stain Kit (Invitrogen, Waltham, MA, USA), respectively.

### RNA-seq and data availability

Total RNA was extracted via the RNeasy Mini Kit (Qiagen) after cell lysis. Libraries for transcriptome analysis were prepared via the Smart-seq2 protocol [32] starting from 2 ng of total RNA and then sequenced on the Illumina NextSeq 500 in single-read mode (1 × 75 cycles) at the Centro Piattaforme Tecnologiche of the University of Verona. This study also utilized additional bulk RNA-seq datasets previously generated by Smart-seq2 [5], which were downloaded from the Gene Expression Omnibus database [33] (http://www.ncbi.nlm.nih.gov/geo/) under the accession number GSE164687. The computational analysis was conducted via a previously described bioinformatics pipeline [5], with slight modifications. Gene counts were normalized among various samples via DESeq2 [34], and only genes coding for proteins and long noncoding RNAs were retained for downstream analysis. DEGs were identified via DESeq2, with a selection parameter adjusted *P* value lower than 0.01 and a likelihood ratio test 36. PCA was performed on the DEGs via the Bioconductor/R package pcaExplorer v.2.10.0. To infer the developmental path of neutrophils, we used hierarchical clustering with Euclidean distance and Ward’s method, followed by optimal leaf ordering via the R package seriation, v.1.2-9 [35]. The raw datasets generated in this study have been submitted to the Gene Expression Omnibus (GEO) database [33] (http://www.ncbi.nlm.nih.gov/geo.) and are available under the accession number GSE274002.

### Measurement of O_2_^−^ production, phagocytosis, and cytokine release

Freshly isolated HD neutrophils and cells generated from NCP3s, NCP4s, NCP5s, and NCP6s cultured for 5 d with SFGc were washed and then resuspended at 2.5 × 10^5^/ml in HBSS supplemented with 10% FBS containing 1 mM CaCl_2_ and 5 mM glucose. O_2_^−^ production in response to 20 ng/ml phorbol-myristate acetate (PMA, Sigma‒Aldrich) was determined via a cytochrome C reduction assay [36]. Phagocytosis was evaluated by incubating 2.5 × 10^4^ cells/100 μl in the presence of 20 μg/ml unopsonized zymosan particles in flat-bottom 96-well tissue culture plates at 37 °C (as well as at 4 °C as a control) [5]. After 30 min, phagocytosis was terminated by the addition of excess cold HBSS, after which the cells were recovered and centrifuged via Cytospin onto slides to be stained via the May-Grunwald Giemsa procedure and analyzed via an OLYMPUS BX51 microscope equipped with a U-RFL-T camera. For cytokine production, 2.5 × 10^4^ cells/100 μl of RPMI 1640 medium (Thermo Fisher Scientific, Waltham, MA, USA) containing 10% FBS were incubated at 37 °C in the presence or absence of 1μg/ml ultrapure LPS (*E. coli* 0111:B4 strain, InvivoGen). After 20 h, the resulting cell-free supernatants were collected and analyzed via commercial enzyme-linked immunosorbent (ELISA) kits: CXCL8 (Mabtech, Nacka Strand, Sweden), IL-1ra and BAFF (R&D System, Minneapolis, MN, USA). The lowest detection limits for these ELISAs are 7.8 pg/ml for CXCL8 and 39.1 pg/ml for IL-1ra and BAFF.

### Immunocytochemistry (ICC) and immunohistochemistry (IHC)

Sorted NCPs and PMs were spotted on polarized slides and immediately fixed with 95% ethanol for immunostaining with anti-elastase mAb (1:200 dilution, clone NP57, Agilent Technologies), anti-αdefensin mAb (dilution 1:70, clone H-2, Santa Cruz Biotechnology) and anti-arginase-1 mAb (1:100 dilution, clone SP156, Cell Marque), as previously described [5]. Immunostained slides were photographed via a DP-73 Olympus digital camera and mounted on an Olympus BX60 microscope (×600 magnification, square side: 20 μm), and from ten to forty NCP5s, NCP6s, and PMs were scored as strongly positive (= strong cytoplasmic reactivity), weakly positive (= only a few granules stained/weak cytoplasmic reactivity), or negative (= no reactivity). FFPE bone marrow samples from HDs (*n* = 3) and CP-CML patients (*n* = 3) were retrieved from the archive of U.O. Anatomia Patologica, Spedali Civili di Brescia, and sequentially immunostained for CD45RA (clone 4KB5, dilution 1:90, Abcam, #ab755), CD34 (clone QBEND/10, dilution 1:100, Leica Biosystems, #END-L-CE), IRF8 (clone V3GYWCH, dilution 1:200, Invitrogen, #53-9852-82), MPO (pAb, dilution 1:4000, Agilent Technologies, #A0398), CD66B (clone G10F5, dilution 1:200, BioLegend, #305102) and DEFA (clone H-2, dilution 1:3000, Santa Cruz Biotechnology, #sc-390796). Briefly, 4-micron sections were cut and mounted on charged slides. Then, the sections were kept in the oven at 60 °C for 10 min before being subjected to immunostaining. The sections were deparaffinized, and methanol plus H_2_O_2_ (0.03%) was used to quench endogenous peroxidase activity. Antigen retrieval was performed before primary antibody incubation and was different for each antibody according to evidence from decalcified BM tissue; microwaves 3 × 5 min at 750 W (CD66b, CD34, DEFA), 2 × 5 min at 750 W (MPO), 2 × 5’ at maximum Watt and 3 × 5’ at 750 W (CD45RA) or a 98 °C water bath for 40’ (IRF8) were used in different buffers (pH 6.0 citrate for CD66b and pH 8.0 EDTA for the other buffers). Novolink polymer (Leica Microsystems) followed by 3-Ammino-9-etil carbazole (AEC) as a chromogen was used to reveal the reaction. Once the first immunostaining was terminated, the slides were counterstained with H&E, mounted and digitalized (Aperio Scanscope CS, Leica Microsystems). The coverslip was then removed, and the slides were placed in alcohol (30 min to overnight), after which they were allowed to clear (AECs are alcohol soluble), and a new stain was obtained after antigen retrieval was performed. The heat treatment terminates cross-reactivity because of the use of primary antibodies raised from the same species [37].

### Statistical analysis

The data are expressed as the means ± s.e.m.s. Statistical evaluation was performed via one-way analysis of variance (ANOVA) followed by Tukey’s post hoc test, 2-way ANOVA followed by Tukey’s post hoc test, or the Mann‒Whitney test. *P* values < 0.05 were considered statistically significant. Statistical analysis was performed with GraphPad Prism version 9 software (GraphPad Software, La Jolla, CA, USA).

### Study approval

Human samples were obtained following informed written consent by HD, CP-CML, mastocytosis and alloHSCT patients. The study was approved by the Ethics Committee of the Azienda Ospedaliera Universitaria Integrata di Verona (Italy) (#CMRI/55742).

## Supplementary information

Supplemental Material

Supplemental Table 3
