# Supplementary material for: Uncovering two neutrophil-committed progenitors that immediately precede promyelocytes during human neutropoiesis
Source: Cell Mol Immunol. 2025 Feb 13;22(3):316–29. doi: 10.1038/s41423-025-01259-w (PMC11868371; doi:10.1038/s41423-025-01259-w)
Supplement: Supplementary file 1 — Supplemental Material [file 41423_2025_1259_MOESM1_ESM.docx]

Supplementary Material for

**Uncovering two neutrophil-committed progenitors that immediately precede the promyelocytes during human neutropoiesis**

Ilaria Signoretto,^1*^ Federica Calzetti,^1*^ Giulia Finotti,^2*^ Silvia Lonardi,^3^ Camillo Balanzin,^1^ Francisco Bianchetto-Aguilera,^1^ Sara Gasperini,^1^ Elisa Gardiman,^1^ Monica Castellucci,^3^ Anna Russignan,^4^ Massimiliano Bonifacio,^4^ Antonio Sica,^5^ William Vermi,^2^ Cristina Tecchio,^4^ Patrizia Scapini,^1^ Nicola Tamassia,^1^ and Marco A. Cassatella^1^

^1^Department of Medicine, Section of General Pathology, University of Verona, Verona, Italy.

^2^Centro Piattaforme Tecnologiche, University of Verona, Verona, Italy.

^3^Department of Molecular and Translational Medicine, Unit of Pathology, University of Brescia, Brescia, Italy.

^4^Department of Engineering for Innovation Medicine, University of Verona, Verona, Italy.

^5^Department of Pharmaceutical Sciences, University of Piemonte Orientale 'A. Avogadro', Novara // Humanitas Clinical and Research Center, Rozzano, Italy.

^*^these three authors contributed equally to this work

**This file includes:**

Figures S1, S2, S3 and S4

Tables S1 and S2

**Other Supplementary Material for this manuscript includes the following:**

Table S3

***
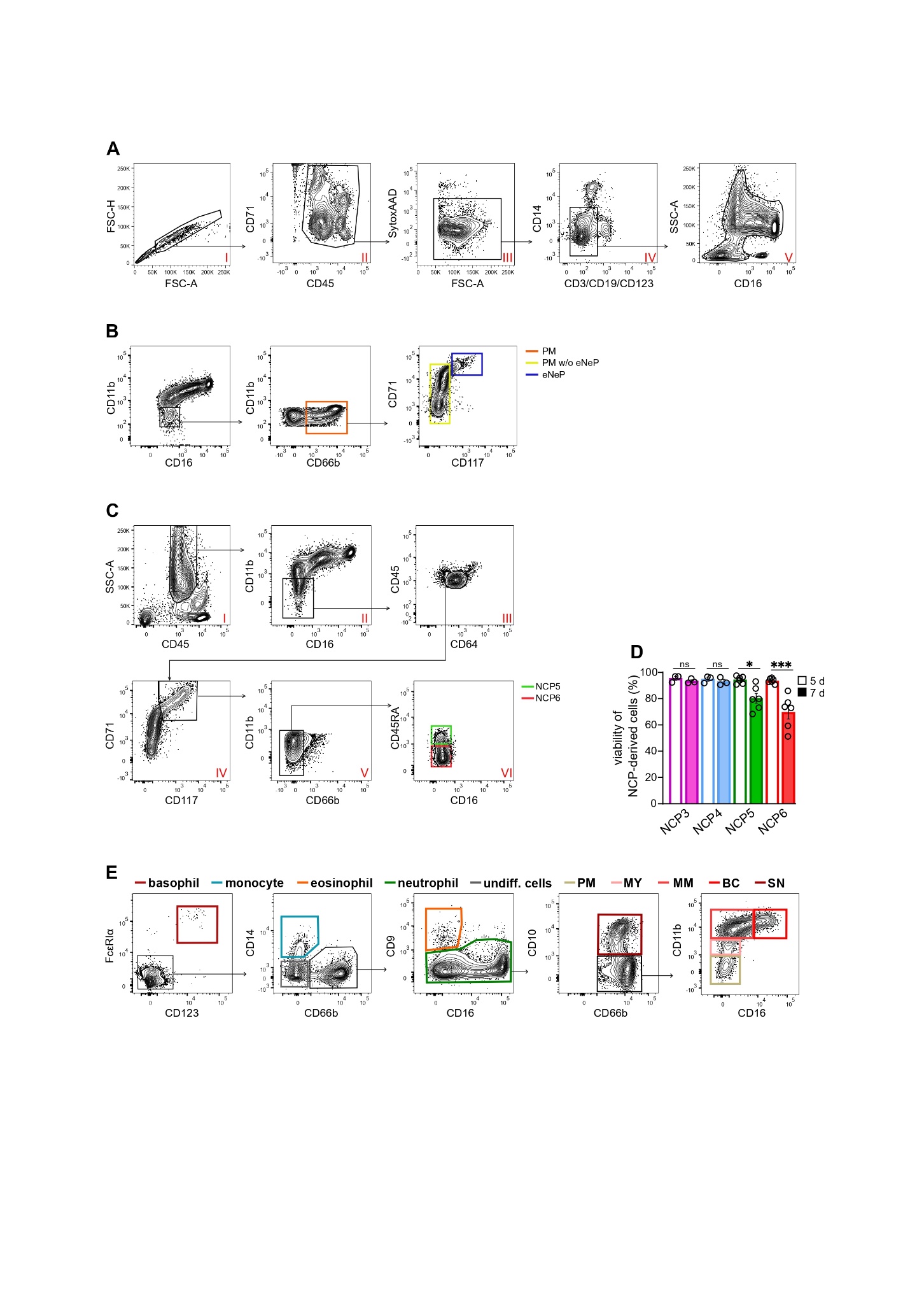
***

**Figure S1. Gating strategy to identify lineage-positive cells, PMs, NCP5s and NCP6s within human BM-LDCs. (A)** Flow cytometry gating strategy selecting singlet cells (panel I), CD45^+^cells (panel II) and live cells (panel III) to exclude CD14^+^monocytes, CD3^+^/CD19^+^lymphocytes, CD123^+^DCs (IV) and CD16^+^NK cells (V), within BM-LDCs. **(B)** Plot representing the identification of CD66b^+^CD11b^-^CD16^-^PMs within the CD11b^-^CD16^-^cells. **(C)** Plots displaying the flow cytometry gating strategy to sort NCP5s (light green gate) and NCP6s (red gate). BM-LDCs were sequentially gated first as SSC^hi^CD45^+^cells (panel I) and then as CD11b^-^CD16^-^cells (panel II), while CD45^+^CD64^br^monocytes and CD45^+^CD64^-^eosinophils (panel III) were excluded by the analysis. Then, within the CD117^+^CD71^+^cell population (panel IV), CD66b^-^cells (panel V) were selected and finally displayed based on their CD45RA expression to identify NCP5s and NCP6s (panel VI). **(D)** Bar graph depicting the percentage of live cells generated by NCP3s, NCP4s, NCP5s and NCP6s treated with SFGc for 5 and 7 days (data represent mean ± s.e.m., n=3 for NCP3s and NCP4s; n=6 for NCP5s and NCP6s). Statistical analysis was performed using Two-way ANOVA and Tukey post hoc test. * = p<0.05, ** = p<0.01, *** = p<0.001. **(E)** Flow cytometry gating strategy to identify basophils (bordeaux gate), monocytes (light blue gate), eosinophils (orange gate) and undifferentiated cells (grey gate), as well as PMs (beige gate), MYs (pink gate), MMs (light red gate), BCs (red gate) and SNs (dark red gate) within the CD45^+^ BM-LDCells.


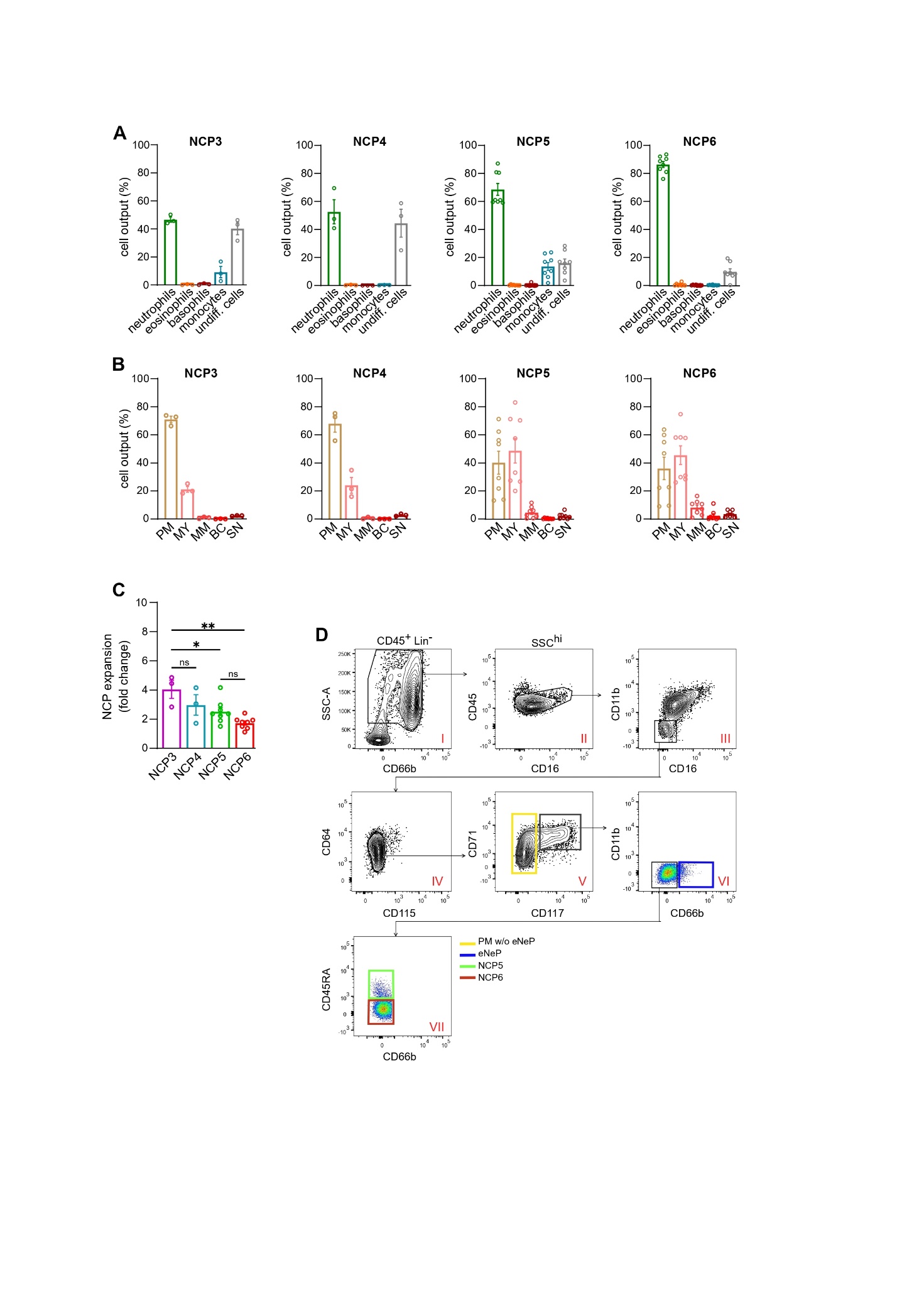


**Figure S2. Differentiation capacity by SFG-treated NCP3s, NCP4s, NCP5s and NCP6s. A)** Bar graphs reporting the percentage of CD66b^+^cells (green contour, mean ± s.e.m referred to the total CD45^+^cells, n=3 for NCP3s and NCP4s; n=8 for NCP5s and NCP6s), eosinophils (orange contour), basophils (bordeaux contour), monocytes (light blue contour) and undifferentiated cells (grey contour) derived from NCP5s and NCP6s treated with SFG for 5 days. **B)** Bar graphs showing the percentage of CD66b^+^PMs (beige contour), MYs (pink contour), MMs (light red contour), BCs (red contour) and SNs (dark red contour) derived from NCP3s, NCP4s, NCP5s and NCP6s treated for 5 days with SFG (mean ± s.e.m., n=3 for NCP3s and NCP4s; n=8 for NCP5s and NCP6s). **C)** Bar graph displaying the fold expansion of NCP3s, NCP4s, NCP5s and NCP6s treated with SFG for 5 days (mean ± s.e.m., n=3 for NCP3s and NCP4s; n=8 for NCP5s and NCP6s). Statistical analysis was performed using one-way ANOVA and Tukey post hoc test. * = p<0.05, ** = p<0.01, *** = p<0.001. **D)** Flow cytometry strategy showing the detection of NCP5s (light green gate), NCP6s (red gate), eNePs (blue gate) and PMs w/o eNePs (yellow gate) in BM-LDCs of reconstituting BM (at day +21) from patients undergoing allogeneic hematopoietic stem cell transplantation. A representative experiment out of 3.


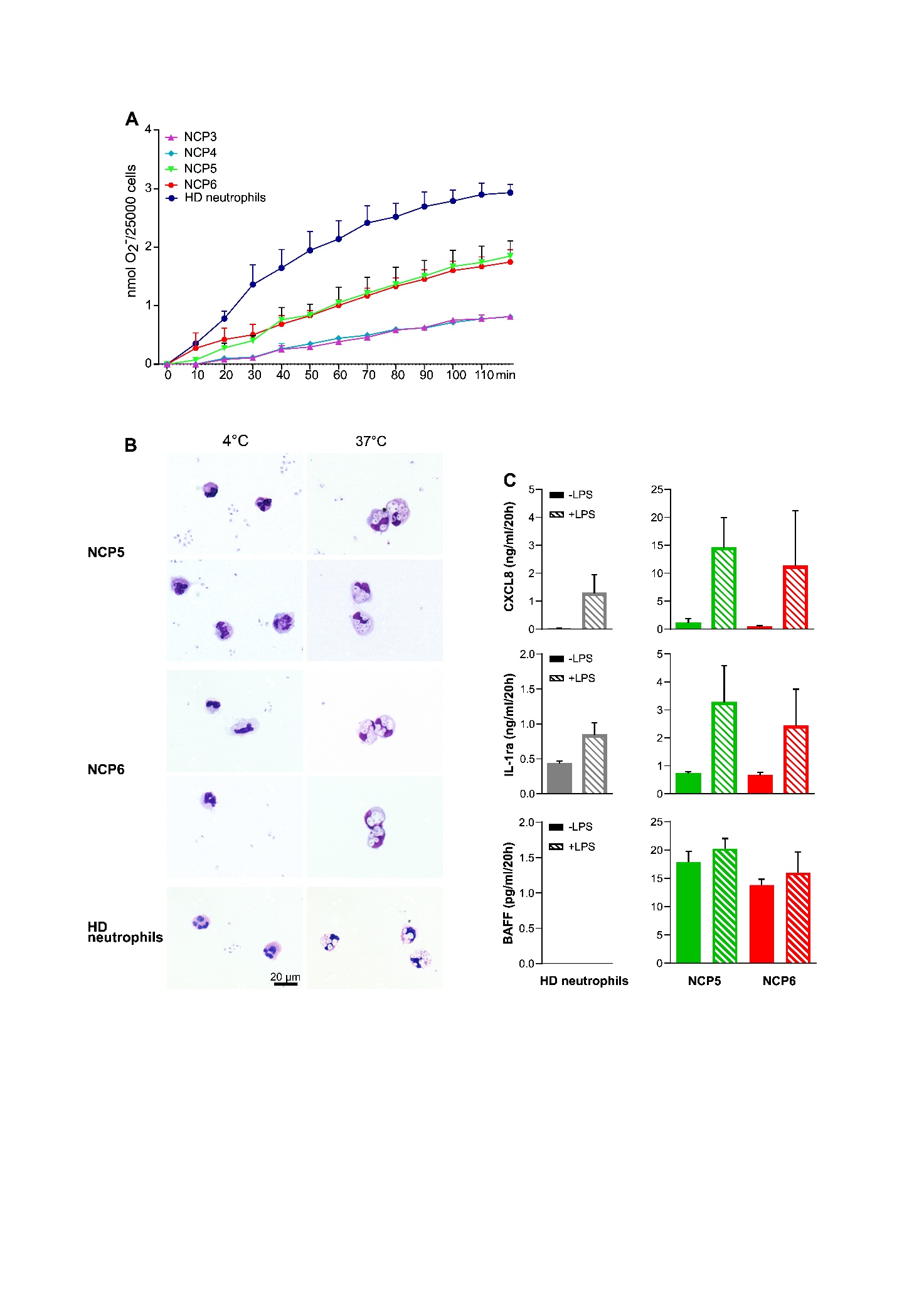


**Figure S3. Functional evaluation of the CD66b^+^cells derived from NCP5s and NCP6s. A)** Kinetics of O_2_^-^ production by either CD66b^+^cells derived from NCP3s (magenta line), NCP4s (light blue line), NCP5s (light green line) and NCP6s (red line) cultured for 5 days with SFGc, or HD neutrophils, stimulated with 20 ng/ml PMA for up to 120 min. Values represent the mean ± s.e.m. (n=3). **B)** Representative cytospin fields showing HD neutrophils and CD66b^+^cells derived from NCP5s and NCP6s phagocytosing unopsonized zymosan particles at 37° (right panels), but not at 4° (left panels). **C)** Histograms displaying the levels of CXCL8/IL-8, IL-1RA and BAFF measured in supernatants collected from CD66b^+^cells derived from NCP5s and NCP6s, as well as from HD neutrophils, incubated with LPS for 20 h. Values represent the mean ± s.e.m. (n=3).


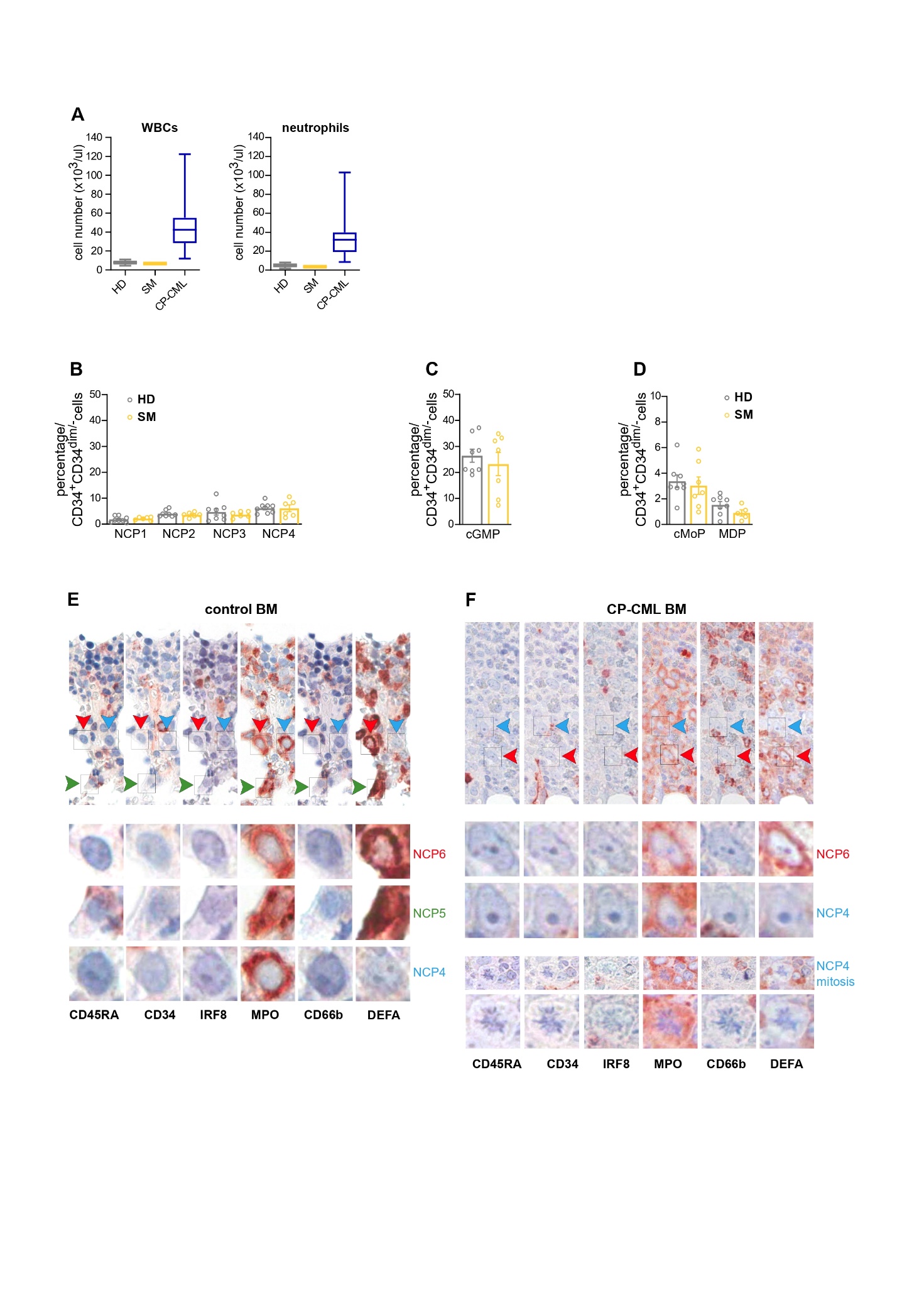


**Figure S4. Counts of peripheral neutrophil counts, neutrophil progenitors in BM-LDCs of SM patients, and immunohistochemistry data from BM-LDCs of HDs and CP-CML patients. (A)** Box plots displaying the number of white blood cells (WBCs) (left panel) and neutrophils (right panel) per microliter from HDs (grey box), SM patients (light yellow box), and CP-CML patients (blue box). **(B)** Bar graph showing the frequency of NCP1s, NCP2s, NCP3s and NCP4s in narrower area defined as CD34^+^/CD34^dim/-^cells in HDs (n=8, grey contour) as compared to SM patients (yellow contour, n=6). Data are represented as mean ± s.e.m. and Mann-Whitney test was performed. **(C,D)** Bar graph showing the frequency of cGMPs, cMoPs and MDPs in CD34^+^/CD34^dim/-^cells of HDs (grey contour, n=8) as compared to SM patients (yellow contour, cGMPs and cMoPs, n=7; MDPs, n=5). Data are represented as mean ± s.e.m. and t-test was performed. **(E,F)** Magnifications of immunostained sections shown in Figure 6, panels E and F. Examples of NCP4s, NCP5s and NCP6s from control BM, and of NCP4s and NCP6s from CP-CML were digitally magnified. Squares and arrows (turquoise for NCP4, green for NCP5 and red for NCP6) identify the magnified cell. An example of mitotic NCP4s is also shown.

**Table S1. Information on healthy donors (HDs) and patients with chronic phase-chronic myeloid leukemia (CP-CML) and systemic mastocytosis (SM).**

|  | **donor** | **male/female** | **age (years)** | **average age (years)** |
| --- | --- | --- | --- | --- |
|  | 1 | M | 25 |  |
|  | 2 | F | 28 |  |
|  | 3 | F | 22 |  |
|  | 4 | M | 20 |  |
|  | 5 | F | 22 |  |
|  | 6 | F | 38 |  |
|  | 7 | F | 27 |  |
|  | 8 | M | 26 |  |
| **HDs** | 9 | M | 23 | 28 |
|  | 10 | M | 24 |  |
|  | 11 | M | 21 |  |
|  | 12 | F | 36 |  |
|  | 13 | F | 24 |  |
|  | 14 | F | 25 |  |
|  | 15 | M | 26 |  |
|  | 16 | F | 66 |  |
|  | 17 | M | 25 |  |
|  | 18 | M | 31 |  |
|  | 19 | M | 32 |  |
|  | 20 | M | 26 |  |
|  | 21 | F | 27 |  |
|  | 1 | M | 38 |  |
|  | 2 | M | 73 |  |
|  | 3 | M | 64 |  |
|  | 4 | F | 71 |  |
|  | 5 | F | 63 |  |
|  | 6 | F | 69 |  |
|  | 7 | M | 19 |  |
|  | 8 | F | 68 |  |
| **CP-CML** | 9 | M | 62 | 53 |
| **patients** | 10 | M | 45 |  |
|  | 11 | M | 48 |  |
|  | 12 | F | 42 |  |
|  | 13 | M | 38 |  |
|  | 14 | F | 58 |  |
|  | 15 | M | 45 |  |
|  | 16 | M | 50 |  |
|  | 1 | M | 68 |  |
|  | 2 | M | 49 |  |
| **systemic** | 3 | F | 27 |  |
| **mastocytosis** | 4 | F | 64 | 52 |
| **patients** | 5 | F | 64 |  |
|  | 6 | F | 38 |  |
|  | 7 | M | 56 |  |

**Table S2. List of monoclonal antibodies used for the flow cytometry experiments**

| Fluorophore | Antibody (clone) | Cat. Number +  Rrid | Manufacturer |
| --- | --- | --- | --- |
| BUV395 | CD45 (HI30) | 563792; AB_2869519 | BD Biosciences |
| BUV496 | CD3 (UCHT1) | 612941; AB_2916883 | BD Biosciences |
|  | CD19 (HIB19) | 741141; AB_2870719 | BD Biosciences |
|  | CD123 (6H6) | 751836; AB_2875807 | BD Biosciences |
|  | CD38 (HIT2) | 612947; AB_2916885 | BD Biosciences |
| BV421 | CD117 (104D2) | 2166080; AB_10896056 | Sony Biotechnology |
|  | CD10 (HI10a) | 312218; AB_2561833 | Biolegend |
|  | CD66b (G10F5) | 562940; AB_2737906 | BD Biosciences |
|  | CD125 (A14) | 743927; AB_2741855 | BD Biosciences |
|  | FceR1α (AER-37) | 747787; AB_2872251 | BD Biosciences |
| BV510 | CD45 (HI30) | 304036; AB_2561940 | Biolegend |
| VioGreen | CD45RA (REA562) | 130-113-369; AB_2726139 | Miltenyi-Biotech |
| BV570 | CD14 (M5E2) | 2109160; AB_10897803 | Sony Biotechnology |
| BV605 | CD11b (ICRF44) | 562721; AB_2737745 | BD biosciences |
|  | CD15 (W6D3) | 562980; AB_2744292 | BD biosciences |
| BV650 | CD11b (ICRF44) | 301336; AB_2563793 | Biolegend |
|  | CD45RA (HI100) | 304136; AB_2563653 | Biolegend |
| BV786 | CD14 (M5E2) | 563698; AB_2744287 | BD Biosciences |
| AF488 | CD64 (10.1) | 305010; AB_528865 | Biolegend |
|  | CD115 (9-4D2-1E4) | 347312; AB_2566758 | Biolegend |
| FITC | CD66c (B6.2/CD66) | 551775; AB_394249 | BD Biosciences |
| PE | CD115 (9-4D2-1E4) | 347304; AB_2085255 | Biolegend |
|  | CD9 (REA1071) | 130-118-807; AB_2733845 | Miltenyi-Biotech |
|  | CD64 (10.1) | 2125040; AB_314491 | Sony Biotechnology |
| PE-CF594 | CD10 (HI10a) | 312228; AB_2565878 | Biolegend |
| PerCP/Cy5.5 | CD16 (3G8) | 2110140; AB_893262 | Sony Biotechnology |
| PE/Cy7 | CD123 (REA918) | 130-115-266; AB_2726970 | Miltenyi-Biotech |
|  | CD49d (REA545) | 130-125-506; AB_2819757 | Miltenyi-Biotech |
|  | CD45RA (REA1047) | 130-117-746; AB_2733237 | Miltenyi-Biotech |
|  | CD11b (ICRF44) | 301322; AB_830644 | BioLegend |
| APC | CD34(AC136) | 130-113-176; AB_2726003 | Miltenyi-Biotech |
|  | CD19(HIB19) | 302212; AB_314242 | Biolegend |
| AF700 | CD66b(G10F5) | 2125570; AB_2566037 | Sony Biotechnology |
| APC/Cy7 | CD71(CY1G4) | 2270550; AB_2563116 | Sony Biotechnology |
